# Proteomic and transcriptomic signatures of cytoskeletal remodeling during morphogenesis in the basal metazoan *Halisarca dujardinii* (Porifera)

**DOI:** 10.3389/fcell.2026.1829393

**Published:** 2026-06-10

**Authors:** Alexei A. Kotov, Alexander D. Finoshin, Kim I. Adameyko, Vasiliy M. Zubarev, Anton V. Burakov, Marat S. Sabirov, Kirill V. Mikhailov, Rustam H. Ziganshin, Nikolay G. Gornostayev, Pavel A. Erokhov, Margarita A. Ezhova, Elena I. Shagimardanova, Victor S. Mikhailov, Oksana I. Kravchuk, Yulia V. Lyupina

**Affiliations:** 1 N. K. Koltsov Institute of Developmental Biology, Russian Academy of Sciences, Moscow, Russia; 2 Center for Bio- and Medical Technologies, Skolkovo Institute of Science and Technology (Skoltech), Moscow, Russia; 3 A. N. Belozersky Institute of Physical and Chemical Biology, Lomonosov Moscow State University, Moscow, Russia; 4 Life Improvement By Future Technologies Institute, Moscow, Russia; 5 A. A. Kharkevich Institute for Information Transmission Problems, Russian Academy of Sciences, Moscow, Russia; 6 Shemyakin and Ovchinnikov Institute of Bioorganic Chemistry, Russian Academy of Sciences, Moscow, Russia

**Keywords:** cytoskeleton, cytoskeleton remodeling, *Halisarca dujardinii*, Porifera, proteasome, proteome, redox signaling

## Abstract

Sponges are capable of rebuilding entire functional organisms from dissociated somatic cells. This feat demands radical cytoskeletal reprogramming whose molecular logic remains obscure. Using the Arctic demosponge *Halisarca dujardinii*, we mapped molecular signatures across free-swimming larvae, sessile adults, and early cell aggregates. Integrating RNA-seq, single-cell deconvolution, DIA-LC-MS proteomics, native complex fractionation, live-cell proteasome imaging, and immunofluorescence, we identified stage-associated profiles of cytoskeletal proteins and post-translational modifications (PTMs). Larvae enrich non-classical myosins (Myosin-9/15) and dynamic microtubules for motility; adults deploy spectrin scaffolds and deacetylated microtubules for stability; cell aggregates shift toward microtubule-based transport with targeted proteolysis and stage-specific PTMs on divergent actin HdA6 and ferritin. Live imaging shows ubiquitin-independent 20S proteasome activity at cell contacts in forming aggregates, while mass spectrometry reveals coordinated methionine oxidation and ubiquitination events coinciding with cytoskeletal remodeling. Although bulk data cannot fully separate cell-type composition from intracellular reprogramming, the multi-layered signatures suggest a tightly regulated, stage-specific program. Together, these findings establish a correlative framework linking cytoskeletal architecture, redox-sensitive PTMs, and proteostasis within sponge development and early reaggregation stage. We propose that conserved structural networks are chemically tuned to enable morphological plasticity in early-branching metazoans - a foundation for future functional studies of multicellular morphogenesis.

## Introduction

Cytoskeletal reorganization accompanies regeneration in a wide variety of animals, but its mechanisms can differ fundamentally. In planarians (Platyhelminthes), microtubules regulate the expression of key factors in the muscles used to form the blastema during regeneration, but actin-mediated motility is not completely suppressed (Wenemoser et al., 2012; Anderson and Petersen, 2025). In Hydra (Cnidaria), remodeling of the actin cortex occurs during budding and regeneration, and microtubules are involved in maintaining polarity determined by signaling pathways (Bosch et al., 2010; Livshits et al., 2017; Wang et al., 2020). During limb regeneration in the axolotl (Chordata), blastema cell migration is carried out mainly by actin-dependent mechanisms, although microtubules are involved in the transport within the growing limb bud, they do not completely replace actin-dependent motility (Gardiner et al., 1986; McCusker et al., 2015; Seifert and Muneoka, 2018).

Sponges (Porifera), one of the earliest-branching metazoan lineages, have captivated biologists for over a century with their extraordinary capacity to rebuild new functional organisms from dissociated cells - a phenomenon first demonstrated in Wilson’s reaggregation experiments (Wilson, 1907). This whole-body regenerative process is far from monolithic. Crucially, not all sponge species can reassemble new functional bodies from cellular aggregates, suggesting that this morphological flexibility extends well beyond injury repair, and regeneration competence is an adaptive trait shaped by ecological pressures (Ereskovsky et al., 2021). Branching demosponges like *Raspailia ramosa* sculpt forms within hydrodynamics, simplifying architecture in heavily silted habitats rather than elaborating structures for sediment shedding (Bell et al., 2002). The tropical sponge *Anthosigmella varians* dynamically modulates spicule density that fortifying its skeleton under predation threat while reducing mineral investment in safer waters to conserve energy (Hill and Hill, 2002). During *Hymeniacidon heliophila* regeneration, pinacocytes and archaeocytes migrate with purposeful coordination while differentiated cells undergo transdifferentiation to rebuild tissue boundaries (Coutinho et al., 2017). *Oscarella lobularis* (cl. Homoscleromorpha) is capable to form the clonal buds *in vitro*, which regenerate after damage and complete dissociation into the cells (Rocher et al., 2024). In calcareous sponge *Sycon ciliatum*, regeneration partially recapitulates post-larval development, reactivating conserved molecular pathways to reestablish body axes and tissue architecture (Soubigou et al., 2020). Beneath these visible transformations lies cellular reprogramming of remarkable sophistication. Transcriptomic surveys across Porifera reveal deep conservation of developmental toolkits (Wnt, TGF-β, Notch, Hedgehog) alongside striking lineage-specific innovations in cytoskeletal regulation (Riesgo et al., 2014; Gaiti et al., 2017; Borisenko et al., 2019; Melnikov et al., 2026). Particularly striking is the facultative microtubule organization in certain demosponges: while most eukaryotes maintain stable interphase microtubule arrays, *Halisarca dujardinii* (cl. Demospongiae) dynamically assembles cytoplasmic microtubules only during regeneration and, keeping them largely absent in sessile adults (Golyshev et al., 2024). This contrasts sharply with calcareous sponges where microtubule networks remain constitutively present, highlighting how cytoskeletal strategies reflect adaptive solutions to distinct ecological niches (Skorentseva et al., 2025).

The Arctic demosponge *H. dujardinii,* lacking mineralized spicules and exhibiting exceptional reaggregation capacity, provides a compelling model to a unique opportunity to trace the cytoskeletal rearrangements during the transitions. Its life cycle spans free-swimming larvae (developing in June) deploying dynamic microtubule networks for locomotion, sessile adults exist throughout the annual cycle (Ereskovsky, 2000). The *in vitro* reaggregation of dissociated somatic cells from *H. dujardinii* captures the earliest phase of a broader regenerative process, because the early cell aggregates retain the capacity to develop into a juvenile sponge, and offer a straightforward and reproducible model for studying the molecular triggers of body assembly (Melnikov et al., 2026). Despite decades of fascination with sponge regeneration, the molecular mechanisms driving cytoskeletal remodeling remain poorly understood. In particular, how PTMs repurpose existing protein scaffolds without *de novo* synthesis is still unknown. Moreover, while sponge genomes have been extensively characterized, comprehensive proteomic comparisons across life stages are largely absent. Here, we integrate transcriptomics, proteomics, single-cell RNA-seq deconvolution, and live imaging to trace how *H*. *dujardinii* achieves remarkable morphological plasticity. This work establishes a correlative framework linking transcriptional programs, protein abundance, and PTM dynamics to stage-specific cellular architecture. Together, these patterns suggest that chemical tuning of conserved cytoskeletal networks may have provided an energy-efficient strategy for early metazoans to navigate the transition toward complex multicellularity.

## Materials and methods

The specimens of Arctic sponge *H. dujardinii* were collected in the sublittoral zone of the White sea (2–5 m depth) near the Polar Circle tourist Center (66° 30′N, 33° 08 E) during their reproductive period in late June 2025 (Supplementary Figures S1A–E). The sponges were collected together with their substrate (macroalgae) to preserve the natural microenvironment, and maintained as described earlier (Adameyko et al., 2021). The adult sponges with larvae were placed in an aquarium with seawater, and larvae that left the parent and swam freely were collected. Dissociation of sponge body and collection of cell aggregates were performed as described in detail in the Supplementary methods.

The *in vivo* imaging of aggregating cells of sponge specimens was performed by Zeiss LSM880 confocal microscope, equipped with Zeiss Plan-APOCHROMAT 63х/1.4 Oil DIC objective lens (Zeiss, Oberkochen, Germany) as described previously (Lyupina et al., 2025). Active proteasomes were detected using 1 nM Me4BodipyFL-Ahx3Leu3VS (UbiQbio, Amsterdam, Netherlands) added immediately after the sponge body dissociation at +6 to +8 °C. The aggregating sponge cells were recorded 1 hour after the dissociation by confocal laser scanning microscope Carl Zeiss LSM 880 with excitation and emission parameters 511 and 533 nm, respectively.

To test whether proteasome activity is required for cell reaggregation, the dissociated *H. dujardinii* cells were treated with selective proteasome inhibitor, bortezomib (2.5, 5, and 10 nM) during reaggregation and then assessed the morphological parameters of 24 hpd aggregates, as described in the Supplementary methods.

Electrophoresis of sponge proteins in a native polyacrylamide gel followed by detection of chymotrypsin-like proteasome activity (ChLA) in the gel was performed as described earlier (Adameyko et al., 2021). Extended electrophoresis allowed protein complexes to reach positions in the polyacrylamide gradient according to their masses. The electrophoresis was terminated when two colored and differently charged thyroglobulin markers labelled with Cy3.5 (Lumiprobe, Hunt Valley, MD, United States) reached nearly identical positions in the gel. To visualize proteasome proteolytic activities, 200 mM Na-HEPES buffer, pH 7.5, containing 30 μM fluorogenic substrate N-succinyl-leu-leu-val-tyr-amido-4-methyl coumarin (Suc-LLVY-AMC) (1/20 of gradient gel volume) was applied to the gel surface. The gel was covered by cellulose acetate film and incubated at 37 °C for 20 min. Fluorescence bands in the gel were photographed in a dark room under illumination at 365 nm. To reveal the specificity of signal of proteasome activity the selective proteasome inhibitor Bortezomib at the dose of 5 nM was added to the sample 30 min before the proteasome substrate Suc-LLVY-AMC. The inhibitor concentration was selected based on the survival of cells within its presence (Supplementary Table S1).

To identify the cytoskeletal protein contents and post-translation features of adult sponges, cell aggregates, and larvae were analyzed by LC–MS/MS. The native protein complexes (ferritin complex - 550 kDa band) of *H. dujardinii* body, larvae, and 24 hpd aggregates were isolated from the native gel and analyzed by LC–MS/MS as described previously (Adameyko et al., 2021). The *H*. *dujardinii* body, larvae, 24 hpd cell aggregate lysates were also separated by sodium dodecyl sulfate 10% polyacrylamide gel electrophoresis (SDS-PAGE) electrophoresis, followed by Coomassie blue staining and the bands corresponding to (35–40 and 55 kDa) were analyzed by LC–MS/MS as described earlier (Lyupina et al., 2025). Each group of proteomic samples was represented by at least three biological replicates. To study post-translational modifications of proteins, we used DIA-NN analysis, as described in the Supplementary methods.

Larvae of the Arctic sponge *H. dujardinii* appear only once a year, that gives us a naturally synchronized, developmentally coherent stage that can be cleanly compared to adults sampled at the same time. The proteomic analysis of adult sponge and larvae (three biological replicates per stage) was performed using DIA-LC-MS on an Ultimate 3000 Nano LC system coupled to an Orbitrap Tribrid Lumos mass spectrometer (Thermo Fisher Scientific) as described in detail in the Supplementary methods and Supplementary Table S2. Identification and quantification were performed in DIA-NN v.2.2.0 using a protein database constructed from *H. dujardinii* genome assembly (GCA_054858975.1). Quantitative analysis of protein abundance was performed in Perseus v.2.0.10 (for more details see Supplementary methods). The cell aggregates are formed *in vitro* by dissociating adult sponges, whose physiological state and consequently their molecular composition varies within the seasons. The informative *H. dujardinii* aggregate proteome would require sampling across multiple seasons, which was beyond the scope of this work.

Transcriptome profiling was performed using RNA-seq (BioProject: PRJNA1418124) for three sponge stages: adult sponge tissue, free-swimming larvae, and 24 hpd cell aggregates (three biological replicates per stage) as described in detail in the supplementary methods.

Functional enrichment analysis was performed in STRING (https://string-db.org/) using default parameters as described in the supplementary methods.

Single-cell RNA sequencing (scRNA-seq) data of the cold-water demosponge *H*. *dujardinii* (NCBI SRA database under BioSample accessions SAMN42483027 and SAMN42483028) were generated in our previous study (Lyupina et al., 2025). Data processing and analysis were performed using Seurat v5.0.1, which included read count normalization, selection of variable features, scaling, dimensionality reduction, and cell clustering. Cluster relationships were visualized using Uniform Manifold Approximation and Projection (UMAP). Marker genes for each cluster were identified with the FindAllMarkers function and used to annotate cell identities.

To validate the tubulin localizations in adult (sponge body and 24 hpd cell aggregates) and larval stages, immunofluorescence microscopy was performed with mouse monoclonal against α-tubulin DM-1A (T9026, Sigma-Aldrich, Saint Louis, MO, USA) as described in details in the Supplementary methods. For imaging, a Zeiss LSM900 confocal microscope equipped with Zeiss Plan-APOCHROMAT 63×/1.4 Oil DIC objective lens (Zeiss, Oberkochen, Germany) was used (provided by the Moscow State University Development Program).

Statistical analysis is described in the Supplementary methods. To study the oxidation of methionine, we used Python codes that were created using the Google Colab service and are available on the website (https://colab.research.google.com/drive/1ljzWHx3ypQKZ2tLXPJ-tFFJZ4LARDVs9#scrollTo=qJ93wLyYXlcG).

## Results

**FIGURE 1:**
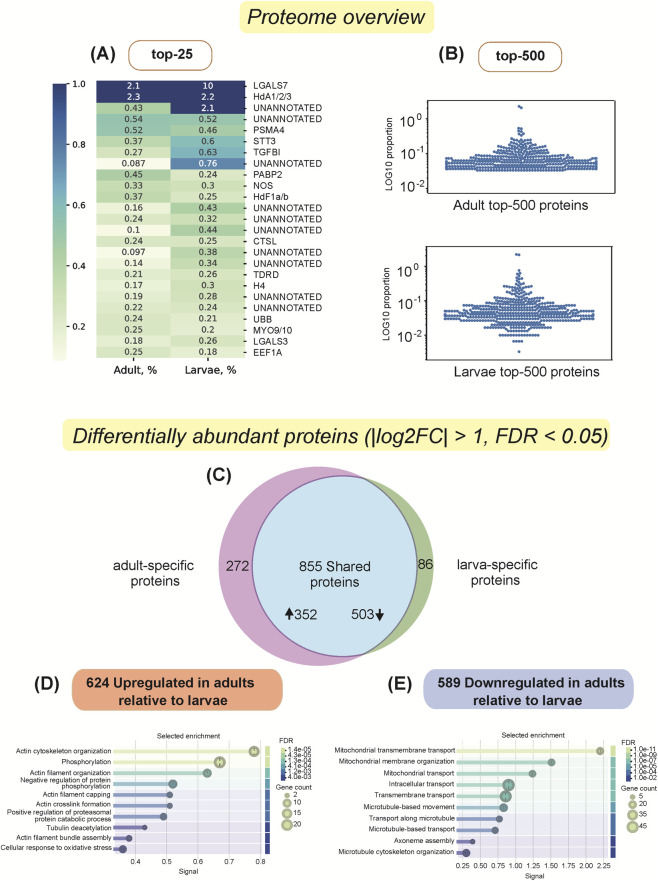
Proteomic analysis of larval and adult *H. dujardinii*. **(A)** The most abundant proteins (top 25) in larvae and adults. *H. dujardinii* proteins: LGALS7 – Galectin 7, HdA1/2/3 – Actin 1/2/3, PSMA4 – 20S proteasome subunit alpha 4, STT3, Protein glycosyltransferase subunit STT3; TGFBI, Transforming growth factor-beta-induced protein; PABP2, Polyadenylate-binding protein 2; NOS, Nitric oxide synthase; HdF1a/b, Ferritin 1a/b; CTSL, Cathepsin L; TDRD, Tudor domain containing protein; H4, H4 histone; UBB, Ubiquitin B; MYO9/10, Myosin class II 9/10; LGALS3, Galectin 3; EEF1A, elongation factor 1 alpha. **(B)** Distribution of relative protein abundance in the top 500 proteins of adults and larvae. Each point represents an individual protein, plotted as log10-transformed proportion. **(C)** Venn diagram showing the overlap between differentially abundant in adults and larvae proteins (|log2FC| > 1, FDR <0.05), including shared and stage-specific proteins. **(D)** Functional enrichment analysis (GO) of proteins upregulated in adults relative to larvae. Gene counts and significance levels (FDR) are indicated for each category. **(E)** Functional enrichment analysis of proteins downregulated in adults relative to larvae.

To understand how Arctic demosponge *H. dujardinii* coordinates cytoskeletal reorganization across its life stages, we integrated transcriptomic and proteomic profiling of adults, larvae, and 24 hpd cell aggregates, asking whether stage-specific morphological transitions are driven primarily by transcriptional reprogramming, post-transcriptional tuning, or a combination of both? The proteomic DIA-LC-MS analysis of naturally synchronized sampling of sponge *H. dujardinii* identified a total of 10,733 proteins (Supplementary Table S3) across larval and adult samples (2,918–10,087 proteins per sample). Principal component analysis (PCA) demonstrated separation of adult and larval samples (Supplementary Figure S2A). Among the 25 most abundant proteins across the developmental stages examined, significant enrichment was observed for the conserved actins HdA1/2/3 and ferritins (HdF1a/b), the 20S proteasome α4 subunit (PSMA4), and several sponge-specific proteins (Figure 1A). Analysis of the 500 most abundant proteins in each proteome (Figure 1B) revealed a core of highly abundant factors involved in fundamental cellular processes, including cytoskeletal organization, the ubiquitin-proteasome system, membrane-associated complexes, histones and their modifying enzymes, and proteins mediating mRNA synthesis, splicing, translation, and cell-cell interactions. In adults, the 500 most abundant proteins were relatively evenly distributed in abundance, consistent with functionally stabilized tissue architecture. In contrast, larval proteomes exhibited a more skewed distribution, dominated by a subset of highly abundant proteins (Figure 1B). Comparative proteomic analysis identified 1,213 proteins with differential expression between larvae and adults (|log_2_FC| > 1, FDR <0.05), of which 272 were found exclusively in adults and only 86 in larvae (Figure 1C; Supplementary Table S4). Of the 855 proteins common to both stages, 352 were elevated in adults and 503 were downregulated compared to larvae. Gene ontology (GO) enrichment analysis of differentially expressed proteins revealed that adults had elevated levels of regulators of actin cytoskeleton dynamics and tubulin deacetylation (Figure 1D), but reduced levels of proteins involved in microtubule cytoskeleton organization and microtubule transport (Figure 1E). Stage-specific proteins were also subjected to GO enrichment analysis (Supplementary Figure S3). The limited number of larval-specific proteins (Supplementary Table S4) precluded robust functional enrichment; however, the RBR-type E3 ligase RNF217 was identified among the unique larval proteins. In contrast, adult-specific proteins were significantly enriched in several biological processes, including negative regulation of phosphorylation (GO:0042326, FDR = 0.02), sensory organ morphogenesis (GO:0090596, FDR <3.4E-4), and embryonic development (GO:0009790, FDR = 0.04). This group also included the HECT-type SMURF E3 ubiquitin ligases associated with the regulation of cell proliferation and DNA repair (Supplementary Figure S3).

**FIGURE 2:**
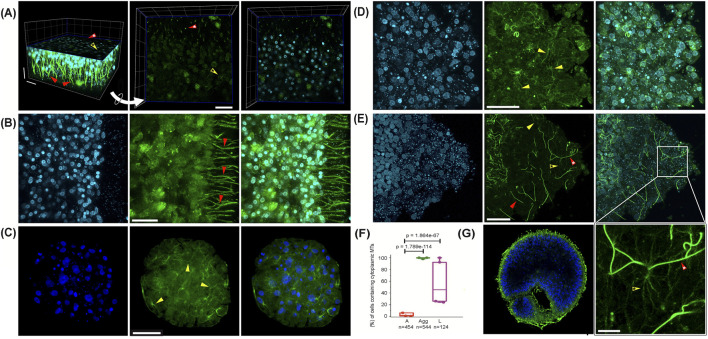
Microtubules in different stages of sponge *H. dujardinii*. Microtubules in adult *H. dujardinii* sponge body **(A,B)**, in the 24 hpd cell aggregate **(C)** and in larvae **(D–F)** stained with antibodies DM1A against tubulin (shown in green). DNA is visualized by staining with Hoechst-33342 solution (shown in blue). The small dots located near the nucleus and stained with Hoechst-33342 at all studied sponge stages are presumably nuclei fragments of another cell types and/or symbionts associated with the sponge *H. dujardinii*. Scale bars are 20 µm in **(A–D)** and for enlarged section of the panel **(E)** shown below is 5 µm. **(A)** Left panel: 3D view of a cross-section of an adult sponge. Right panels: A single focal plane taken parallel to the choanocyte chamber wall deep within the cross-section, oriented toward the viewer. The numerous flagella of choanocytes facing the choanocyte chamber are clearly visible on 3D view (shown by red arrows). An almost complete absence of cytoplasmic microtubules is observed on the single focal plane in the depth of the cross-section, found only in rare individual cells (yellow arrow with an asterisk), along with numerous cross-sectioned choanocyte flagella in the far field of the frame (red arrow with an asterisk). **(B)** Maximum intensity projection of a cross-section of an adult sponge, taken perpendicular to the choanocyte chamber wall. The flagella of choanocytes facing the choanocyte chamber are clearly visible (shown by red arrows), as well as an absence of cytoplasmic microtubules in cells deep within the tissue. **(C)** Maximum intensity projection of a cross-section of the sponge cell aggregate at the 24-h stage. Staining with antibodies to tubulin does not reveal choanocyte flagella, while cytoplasmic microtubules (shown by yellow arrows) are present in many cells, in contrast to adult sponge tissue. **(D,E)** Maximum intensity projections of two different cross-sections of *H. dujardinii.* Larvae revealing a sharp morphological difference between them in the abundance of flagellated cells, which may be either absent **(D)** or present **(E)** within the larval tissue. Choanocyte flagella are indicated by red arrows. Regardless of the presence of flagellated cells, cytoplasmic microtubules are abundantly present in the cells (shown by yellow arrows), often with clearly distinguishable microtubule organizing centers. The area outlined by a square in panel **(E)** is shown below at higher magnification. The red arrow with an asterisk indicating a flagellum and the yellow arrow with an asterisk indicating a cytoplasmic microtubule corresponds to the same arrows above. **(F)** The proportion of *H. dujardinii* cells containing cytoplasmic microtubules (MTs) (%). The Pearson χ^2^ test for 3 × 2 contingency tables used to assess differences between groups. Global test (Chi-square): χ^2^ = 479.17, df = 2, p = 8.88e-105. Post-hoc analysis was performed using Fisher’s exact test for pairwise comparisons between groups. The Bonferroni correction was applied to adjust p-values to account for multiple comparisons. **(G)** General morphology of the *H. dujardinii* larva: cross-section of the larva at its average diameter reveals numerous outward-facing flagella of peripherally located flagellated cells. Scale bar is 100 μm.

Do the stage-specific proteomic signatures of tubulin deacetylation and cytoskeletal regulators we identified lead to differences in cellular architecture? To answer this question, we visualized microtubule organization of adult and larval *H. dujardinii* stages using the anti-tubulin antibody DM1A, which efficiently labels both cytoplasmic microtubules and axonemes (the 20-microtubule core of flagella; Golyshev et al., 2024). Immunofluorescence staining reveals striking differences in microtubule organization between adult sponge *H. dujardinii* and larval stages. In adults, tubulin signal is concentrated almost exclusively in the axonemes of choanocytes (red arrows, Figure 2A), with only sparse and poorly developed cytoplasmic microtubule networks visible in rare cells (yellow arrow, Figure 2B). The 24 hpd cell aggregates do not reveal choanocyte flagella, while cytoplasmic microtubules (shown by yellow arrows, Figure 2C) are present in many cells, in contrast to adult sponge tissue (Figure 2F). By the contrast, larvae exhibit a well-developed cytoplasmic microtubule network in nearly all cells (yellow arrows, Figures 2D–F), irrespective of their flagellar content. Notably, two distinct larval morphotypes were evident: one nearly devoid of flagella (Figure 2D) and another densely packed with them (Figures 2DG) yet both maintained extensive cytoplasmic microtubule arrays.

To determine whether transcriptional shifts or post-transcriptional mechanisms drive *H. dujardinii*’s developmental transitions, we performed RNA-seq analysis of adult sponges, their 24 hpd cell aggregates, and larvae and identified 15,132 out of 28,634 genes with expression levels ≥3 CPM in at least one of these sample groups. PCA demonstrated clear separation of adult, larval, and cell aggregate samples, indicating distinct transcriptional profiles among these conditions (Supplementary Figure S2B). Comparative transcriptomic analysis identified 7,509 differentially expressed genes (DEGs; adjusted p < 0.05, |log_2_FC| > 1) in larvae and only 106 DEGs in 24-h cell aggregates when compared to adult sponges (Supplementary Table S5). We examined in more detail whether the transcription levels of key genes associated with the actin- and tubulin-based cytoskeletal functions differ between larvae, adult sponges, and cell aggregates (Supplementary Figure S4; Supplementary Table S6). The larva exhibits profilin and filamin at minimal levels (mRNA and protein), but has high levels of non-classical myosins. Myosin-15, which attains its highest expression level at the larval stage, is canonically involved in the organization of microvilli and the positioning of primary cilia - structures critical for navigation. Myosin-9, whose expression level also peaks at the larval stage, functions as a “molecular brake,” inactivating Rho-GTPases (RhoA, Rac, Cdc42) and allowing for the immediate halt of local actin remodeling processes. At the protein level, the amount of bicaudal, the key adaptor for dynein and kinesin is reduced in larvae compared to the adults (Supplementary Table S6). The adult radically changes the strategy: profilin, filamin, and spectrin (Supplementary Figure S4; Supplementary Table S6) are expressed at their highest mRNA and protein levels. Interestingly, actin-dependent cell motility is suppressed in cellular aggregates: profilin, filamin, and spectrin mRNA levels reach their minimum levels. The amount of α-tubulin mRNA in cell aggregates is about half that of the adult sponges and even less in the larvae, while the β-tubulin mRNA level does not differ significantly (Supplementary Figure S4; Supplementary Table S6). Mitochondrial proteins LACTB and ABHD10, which form a complex regulating lipid metabolism, were identified in larvae, as well as the E3 ubiquitin ligase RNF217 (RBR family), which is important for mitochondrial homeostasis and the control of reactive oxygen species (Supplementary Figure S3). Taken together, these findings may point to stage-specific rewiring of the cytoskeletal apparatus tuning larvae for active dispersal while equipping adults for the structural stability required by their sessile, filter-feeding lifestyle.

Are the proteomic differences due to changes in sponge stage cellular composition? To link observed transcriptional transitions to underlying cell molecular regulation, we analyzed bulk RNA Seq (adult, larva and cell aggregates) and *H. dujardinii* single-cell RNA-Seq data from our previous study which are publicly accessible via Zenodo (doi.org/10.5281/zenodo.14981466) (Lyupina et al., 2025).

Deconvolution analysis revealed that the studied sponge stages involve some molecular changes and reorganization of cellular composition (Supplementary Figures S5A, B). Importantly, cell populations of cluster 0_2 (corresponds to choanocytes and archaeocytes) are present at all stages, while individual pinacocyte clusters are enriched or depleted. In adults, more than 50% of cells belong to cluster 0_2 along with cluster 7 (corresponds to neuroid pinacocytes). The cluster 7 represents a specialized pinacocyte population enriched in tenascin-like proteins, tyrosine-protein phosphatase Lar, and the T-box transcription factor TBX19 genes likely involved in extracellular matrix formation and maintain the complex tissue architecture of adult sponges. In cell aggregates, the minor cluster 15 with representation of cluster 5 (corresponds to transition choanocytes to pinacocytes) dominates, while larvae consist mainly of cells of cluster 8 (corresponds to transition pinacocytes). The cluster 15 (present predominantly in aggregates) expresses neurocalcin homologues and receptor tyrosine kinases (including a RET proto-oncogene homologue) and exhibits enrichment of collagen-related genes and unique coexpression of receptor signaling genes with peroxidase activity and heme-binding components. Notably, the proportion of cells in clusters 5 and 8 (pinacocytes expressing *HdA6* actin) increases in both larvae and cell aggregates (Supplementary Figure S5C). Interestingly, Myosin 15 is predominantly expressed in cluster 13 associated with pinacocytes in larvae, while Rho GDP dissociation inhibitor (ARHGDI) exhibits increased expression in cluster 15 of cell aggregates (Supplementary Figure S5C) (Yu et al., 2025).

Thus, specific changes in the expression of cytoskeleton-related proteins in certain cell types may influence the functional reorganization required for free-swimming larval forms, cellular aggregates, and filter-feeding adults.

Despite the transcriptional rearrangements we observed, proteomic analysis revealed that some core components remained consistently abundant across all developmental stages. This apparent paradox between dynamic transcriptional regulation and conserved protein abundance suggests that rapid cellular reorganization during sponge metamorphosis may rely not so much on *de novo* protein synthesis as on post-translational mechanisms that dynamically repurpose preexisting cytoskeletal components. The consistent presence of actin HdA1/2/3 and 20S proteasome throughout all developmental stages (Figure 1A), despite significant functional differences between free-swimming larvae and filter-feeding, attached adults, indicated a complex regulatory system operating at the level of protein modification (for example, protein phosphorylation, tubulin deacetylation), not just transcriptional control (Figure 1E; Supplementary Figure S3A). We hypothesized that the rapid reaggregation of dissociated cells can be associated not with the *de novo* synthesis of new components, but with spatiotemporal rearrangement of existing actin networks, and proteasome activity for targeted protein turnover and adhesion. To test this hypothesis, we performed vital confocal microscopy of reaggregating *H. dujardinii* cells using the Me4BodipyFL-Ahx3Leu3VS probe, which detects active forms of the proteasome. This approach was designed to determine whether proteasome activity is localized with filopodia, actin-rich protrusions, and forming cell-cell contacts during the first hours of reaggregation-a time interval when transcriptome analysis revealed minimal changes in gene expression (RNA-seq NCBI Bioprojects PRJNA594150) and highlights the need for chemical rewiring or post-translational regulation of existing cytoskeletal proteins.

**FIGURE 3:**
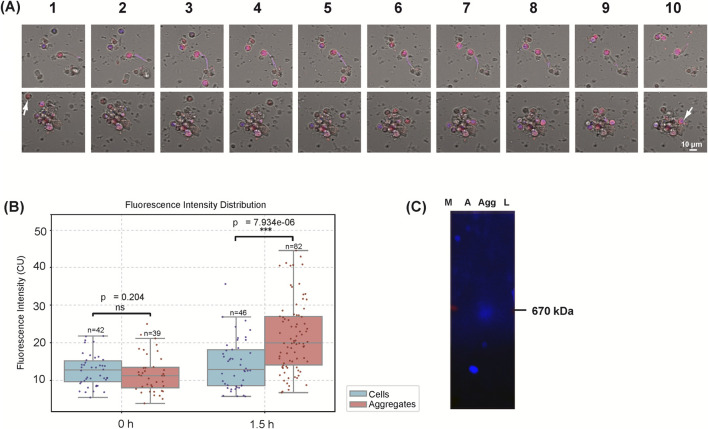
Proteasome functioning in reaggregating sponge *H. dujardinii* cells. **(A)** Confocal images of the reaggregating sponge *H. dujardinii* cells. Two rectangular areas from ten consecutive frames of the Supplementary Video 1 made for 3 h of cell reaggregation in the presence of 1 nM Me4BodipyFL-Ahx3Leu3VS from 18.38 s (frame 1) to 26.19 s (frame 10) are shown. The initial and final positions of first hour of the interacting cells are marked by arrows in A1 and A10 panels. Bar 10 µm. **(B)** Me4BodipyFL-Ahx3Leu3VS fluorescence intensity distribution in cells during reaggregation. The graph shows the intensity of Me4BodipyFL-Ahx3Leu3VS fluorescence (CU) in individual cells (blue) and cells within aggregates (pink) at the beginning and after 1.5 h of reaggregation. The significance of the differences between the ' Cells’ and ‘Aggregates’ group for each time frame was calculated using the Mann-Whitney U test. **(C)** Native PAGE analysis of chymotrypsin-like proteasome activity (ChLA) detected via SLLVY-AMC hydrolysis on a 3%–10% polyacrylamide gradient gel. M, bovine thyroglobulin (670 kDa) molecular weight standard. Forty micrograms of total protein from adult sponge body, 24 hpd aggregates, and larvae were loaded per lane. Full experimental details are provided in Materials and Methods. A–adults, Agg – 24 hpd cell aggregates, L–larvae.

Reaggregation of sponge cells emerged as a highly dynamic process involving active cell migration, frequent intercellular contacts, and progressive aggregate growth. Aggregating cells extended elongated pseudopodia containing active proteasomes (Figure 3), while time-lapse sequences captured sequential attachment of single cells to aggregates via thin filamentous contacts (Figures 3A1–A10; Supplementary Figure S6). A strong fluorescence signal confirmed proteasome activity in the attaching cells (Figures 3A9), at 21.49 s, with cyclic fluctuations in signal intensity during the attachment (Figures 3A5–A10), suggesting dynamic regulation of proteolytic activity during cell–cell adhesion. The intensity of Me4BodipyFL-Ahx3Leu3VS fluorescence in aggregating cells within aggregates after 1.5 h of aggregation was significantly higher than in individual cells (Figure 3B). Thus, monitoring the pulsating and changing fluorescent signals of Me4BodipyFL-Ahx3Leu3VS in real time of aggregation revealed that targeted proteasome proteolysis can acts as an immediate, spatially precise regulator of cytoskeleton remodeling. Concurrently, native PAGE analysis of proteasome complexes from adults, 24 hpd aggregates, and larvae revealed that ChLA was predominantly associated with the core proteasome 20S particle in cell aggregate extracts (Figure 3C). The ChLA was not observed in cell aggregates at the selective proteasome inhibitor bortezomib presence (Supplementary Figure S6C). Moreover, proteasome inhibition by the bortezomib (2.5, 5, and 10 nM), significantly impaired aggregate development in a dose-dependent manner: unit aggregate area increased progressively with increasing bortezomib concentration (p < 0.001 for all comparisons) and circularity and roundness parameters were decreased (p < 0.001, 0.029, and 0.021, respectively) (Supplementary Figure S7). These aggregates fail to progress to later primmorph or juvenile stages (Supplementary Figure S1H-J). These findings demonstrate that ubiquitin-independent 20S proteasome activity is essential for proper cell-cell adhesion, aggregate assembling during early reaggregation in *H. dujardinii*.

Next, we turned to mass spectrometry of native and denatured fractions, mapping post-translational modifications (PTMs) of actin, tubulin, and their regulatory partners in larvae, adults, and regenerating 24 hpd cell aggregates to determine whether *H. dujardinii* achieves its remarkable plasticity through chemical reconfiguration of preexisting protein scaffolds. LC–MS/MS of native and denatured protein fractions revealed stage-specific cytoskeletal organization in sponge *H. dujardinii* (Supplementary Table S7 A-B). The α-tubulin-6 and α-tubulin-7 incorporate into native ferritin complexes exclusively in adults (α-tubulin-6 oxidized at M398). While all α-tubulins appear in SDS fractions across all stages, phosphorylation signatures differ: adults primarily modify S379, whereas larvae and aggregates exclusively target Y312. β-Tubulin shows sequential “oxidative profile” shifts: adults display oxidation at M73 with carbamidomethylation at C12; larvae exhibit deamidation at N337; aggregates transition to oxidation at M330/363 and deamidation at N347 (Supplementary Table S7B; Supplementary Figure S8). Regulatory protein PTMs demonstrate switching: RhoGDI undergoes stage-specific modifications - carbamidomethylation at C106 (adults), deamidation at Q157 (larvae), oxidation at M160 (aggregates). Clathrin (CLTC) appears in native complexes only in larvae and aggregates, acquiring triple modifications (oxidation M1121, deamidation N240, carbamidomethylation C738) specifically in aggregates. Ferritin-actin-tubulin colocalization showed that complexes contain conserved actins (HdA1/2/3) and β-tubulin with overlapping oxidative PTMs (actin: M45/48/228/306/326; tubulin: M73/164/257/293/330) (Supplementary Table S7A-B; Supplementary Figure. S8). Divergent actin HdA6 exhibits stage-specific PTM patterns in cell aggregates: M48 undergoes both oxidation and carbamidomethylation; in larvae it displays maximal PTM diversity including oxidation (M48/51/86/329), carbamidomethylation (C37/56/289), deamidation (Q45), methylation (R43), and ubiquitination (K88/T93) (Supplementary Table S7B). Moreover, the specific markers were detected exclusively in 24 hpd cell aggregates: oxidized ARPC2 (M148/178) and ubiquitinated Ferritin HdF1a/b at S92/C127/T150/K153. Bortezomib exposure (5 µM) during reaggregation shifted ferritin HdF1a/b ubiquitination to residues S13/K19/S118/S120/C127/T150. These data demonstrate that larvae and cell aggregates employ distinct, PTM-driven mechanisms to remodel cytoskeletal proteins, enabling morphological plasticity during development and reaggregation processes.

## Discussion

In this study, we characterize for the first time the proteomes of adult and larval sponges *H. dujardinii* collected simultaneously from the same Arctic habitat, taking advantage of the natural synchronization of sampling. Our analysis reveals extensive cytoskeletal rewiring that underpins both developmental transitions and cell reaggregation in this cold-water demosponge. We demonstrate that larvae, adult sponge, and 24 hpd cell aggregates may employ distinct combinations of cytoskeletal regulators, PTMs, and proteostasis mechanisms to support stage-specific functions: from larval motility to adult structural stability to aggregate-mediated tissue assembly. The proteomic and transcriptomic analyses (Supplementary Table. S6) revealed that larvae exhibit elevated expression of non-classical myosins (myosin-15 and myosin-9), which support ciliary organization and rapid actin remodeling required for swimming (Supplementary Figure S4). Deconvolution of single-cell RNA-seq data localized these myosins primarily to pinacocyte-like cluster 13, which is abundant in aggregates and larvae, but not in adults (Supplementary Figure S5). Notably, myosin-15 expression decreases in cell aggregates (Supplementary Figure S4), suggesting that elevated larval expression reflects genuine stage-specific regulation rather than simply altered cell-type proportions. In contrast, adult sponges display maximal expression of profilin, filamin, and spectrin (Supplementary Figure S4) - proteins that form a dense submembrane scaffold supporting structural stability within the cellularly diverse mesohyl. Confocal immunofluorescence confirmed that adult sponges restrict microtubules largely to stable, acetylated axonemes within choanocytes, whereas larval cells maintain an extensive network of dynamic cytoplasmic microtubules (Figures 2A–D). This architectural shift aligns with the transition from active locomotion to sessile filter-feeding (Nickel, 2006; Borisenko et al., 2019). During cell reaggregation - initiating phase of a juvenile sponge assembling, cells downregulate actin-based motility regulators that can shifts cellular transport toward microtubule-dependent mechanisms. The coordinated molecular switch likely supports the intracellular trafficking demands of tissue reassembly that has been described for demosponge metamorphosis (Sogabe et al., 2016; Riesgo et al., 2022). Beyond proteome and transcriptional regulation (Figure 1; Supplementary Figure S4; Supplementary Table S6), we identified extensive stage-specific PTMs on core cytoskeletal components (Supplementary Table S7 A, B). Despite the high expression of tyrosine kinase Src in adults (Supplementary Table S3), α-tubulins showed no tyrosine phosphorylation, suggesting that adults can actively suppress tyrosine phosphorylation (Figure 1D; Supplementary Figure S3A). The negatively charged phosphate group may prevent the incorporation of tubulin dimers into growing microtubules, thereby promoting their destabilization (Wloga et al., 2017). Notably, deamidation of asparagine 101 in α-tubulins was detected exclusively in cell aggregates, reminiscent of a “molecular clock” mechanism described in mammalian neurons that stabilizes senescent microtubules (Robinson and Robinson, 2001; Adav, 2025). Similarly, oxidation of ARPC2 in aggregates (Supplementary Table S7) suggests localized activation of the Arp2/3 complex for membrane remodeling at nascent cell contacts. The divergent actin HdA6, β-tubulin, ARHGDI, and CLTC display stage-specific dynamic patterns of methionine oxidation, asparagine deamidation, cysteine carbamidomethylation, and ubiquitination (Supplementary Table S7A, B). The findings position HdA6 not merely as a structural element but as a redox-sensitive regulatory node linking oxidative status to cytoskeletal dynamics (Fiaschi et al., 2006; Gellert et al., 2015; Lim et al., 2019; Adameyko et al., 2021). Interestingly, a population of pinacocytes expressing *HdA6* (clusters 5 and 8) is present in both larvae and aggregates (Supplementary Figure S5), but HdA6 has distinctly different PTM signatures in each context (Supplementary Figure S8; Supplementary Table S7), suggesting that chemical “rewiring” can adapts the cytoskeleton to stage-specific processes (Lyupina et al., 2025). Importantly, bulk transcriptomic and proteomic profiling cannot fully distinguish whether these molecular shifts reflect only intracellular chemical reprogramming or cell-type abundance changes. Future studies using higher-resolution approaches, such as spatial transcriptomics, single-cell proteomics, or lineage tracing will be needed to disentangle these contributions. Constitutively high abundance of actin, ferritin, and the α4 subunit of 20S proteasome (PSMA4) across stages (Figure 1A) supposes a conserved proteostasis framework that is differentially engaged during transitions. In humans, PSMA4 was reported to induce metabolic reprogramming by improving oxidative phosphorylation activity in a hypoxia state (Yu et al., 2025). The shifts in proteasome activity during reaggregation coupled with the constitutive high abundance of both actin and 20S proteasome (Figure 3; Supplementary Figure S6) suggest a concerted cellular response, with the actin cytoskeleton providing the framework for cell reassociation, adhesion remodeling, and tissue assembly. This pattern is similar to that observed in mammalian neurons, where the membrane-associated 20S proteasome regulates adhesion without relying on canonical ubiquitin signaling (Ramachandran and Margolis, 2017). In *H. dujardinii*, a burst of proteasome activity during cell reaggregation, along with cell aggregate-specific ubiquitination of ferritin HdF1a/b suggests that targeted protein turnover can manage redox balance within controlled cell remodeling. Bortezomib-induced redistribution of ferritin HdF1a/b ubiquitination sites parallels impaired aggregate compaction (Supplementary Figure S7), suggesting that precise spatiotemporal control of ferritin modification is required for proper morphogenetic progression. Interestingly, cell differentiation in the marine choanoflagellate *Salpingoeca rosetta* depends on utilization of insoluble ferric ions (Leon et al., 2025). The redox-cytoskeletal coupling has been reported in regenerating planarians (Pirotte et al., 2015) and during embryonic development in basal metazoans, underscoring the evolutionary conservation of this strategy (Gellert et al., 2015; Vullien et al., 2025). The stage-specific PTM patterns we describe extend this paradigm by demonstrating how chemical modifications of conserved scaffolds can rapidly reconfigure cellular architecture without requiring *de novo* protein synthesis - a principle increasingly recognized in stress responses across eukaryotes. Notably, the non-overlapping sets of E3 ligases expressed in larvae (RNF217) versus adults (HECT-type) propose distinct ubiquitination programs tailored to stage-specific demands. This observation resonates with recent reports of context-dependent ubiquitin signaling in developmental transitions (Werner et al., 2017), though the precise substrates and regulatory networks in sponges remain to be elucidated.

**FIGURE 4:**
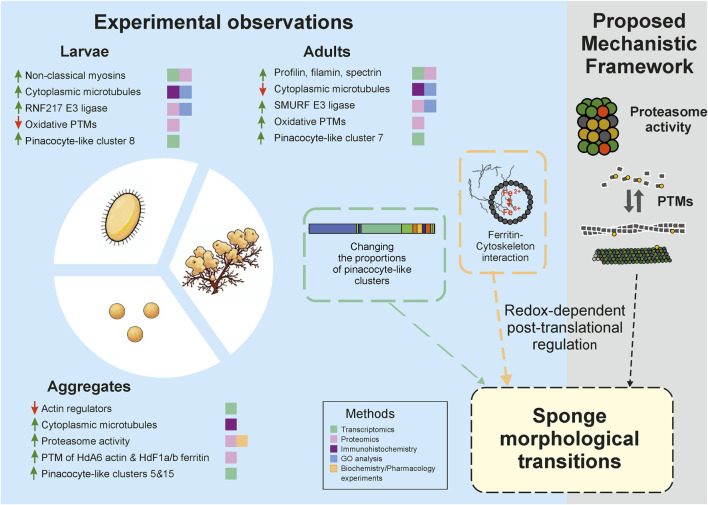
Cytoskeletal rewiring during morphological transitions in the Arctic sponge *H. dujardinii*. Larval cells prioritize motility, deploying extensive cytoplasmic microtubules and non-classical myosins (Myosin-15 and Myosin-9) to support ciliary function and rapid actin remodeling required for swimming (Table S6, Figure S4). This stage is marked by reduced oxidative PTMs, consistent with the high metabolic demands of active locomotion. In contrast, adults favor structural stability: they upregulate profilin, filamin, and spectrin to build a dense submembrane scaffold, restrict microtubules primarily to stable axonemes, and maintain a balanced proteome that supports tissue differentiation within the diverse mesohyl (Table S6, Figure S4). RNA-seq deconvolution revealed the specific changes in the expression of cytoskeleton-related proteins in pinacocyte-like cell types that may influence the functional reorganization required for free-swimming larval forms, cellular aggregates, and filter-feeding adults. The actin-based motility regulators are downregulated in cell aggregates, shifting cellular dynamics toward microtubule-dependent transport coupled to targeted proteolysis and actin HdA6 and ferritin stage-specific PTMs for tissue assembly. Right panel outlines the proposed model for how these transitions are coordinated. The *H. dujardinii* morphological plasticity is driven by a redox-dependent rewiring system: ferritin–actin complexes buffer iron during redox fluctuations (Adameyko et al., 2021), localized 20S proteasome activity mediates targeted protein turnover, and stage-specific PTMs (oxidation, deamidation, and carbamidomethylation) rapidly tune cytoskeletal dynamics without requiring *de novo* synthesis.

We hypothesize how sponge *H. dujardinii* can achieve profound morphological changes through integrated transcriptional, post-translational, and proteostatic regulation across specific-stages (Figure 4).

While we identify stage-specific PTMs in *H. dujardinii* and propose their functional relevance, establishing causality will require targeted chemical proteomics in future studies. The biological variability linked to the reproductive cycle can mask subtler regulatory effects, underscoring the need for seasonal sampling to fully resolve cell aggregate proteomes. Furthermore, studying how sponge-associated bacteria modulate host cytoskeletal regulation through metabolite or signal exchange may reveal how the ubiquitin-proteasome system contributed to the evolutionary emergence of multicellularity.

## Conclusion

In *H. dujardinii*, morphological plasticity emerges from the interplay between the stage-specific expression of pinacocyte-like cells and the dynamic post-translational tuning of conserved structural scaffolds. This “chemical rewiring”, which coordinates redox-sensitive PTMs, targeted proteolysis, and the selective expression of regulatory cytoskeletal proteins may represent an ancient strategy for cellular reorganization. Although direct functional validation is still required, this framework highlights the sophisticated regulatory networks operating at the base of the metazoan tree and suggests that post-translational control of preexisting protein networks likely played a fundamental role in the early evolution of multicellular morphogenesis.

## Data Availability

The datasets presented in this study can be found in online repositories. The names of the repository/repositories and accession number(s) can be found in the article/Supplementary Material.
