# Supplementary material for: Proteomic and transcriptomic signatures of cytoskeletal remodeling during morphogenesis in the basal metazoan Halisarca dujardinii (Porifera)
Source: Front Cell Dev Biol. 2026 Jun 10;14:1829393. doi: 10.3389/fcell.2026.1829393 (PMC13291127; doi:10.3389/fcell.2026.1829393)

Figure S7. Effect of bortezomib on the morphological characteristics of *H. dujardinii* cell aggregates.
The cell aggregates at 24 hpd were formed in the presence of bortezomib at concentrations of 2.5, 5, and 10 nM. Cell area, circularity and roundness were quantified as described in the Supplementary Methods. Scale bar: 100 µm in control, 500 µm in bortezomib samples.

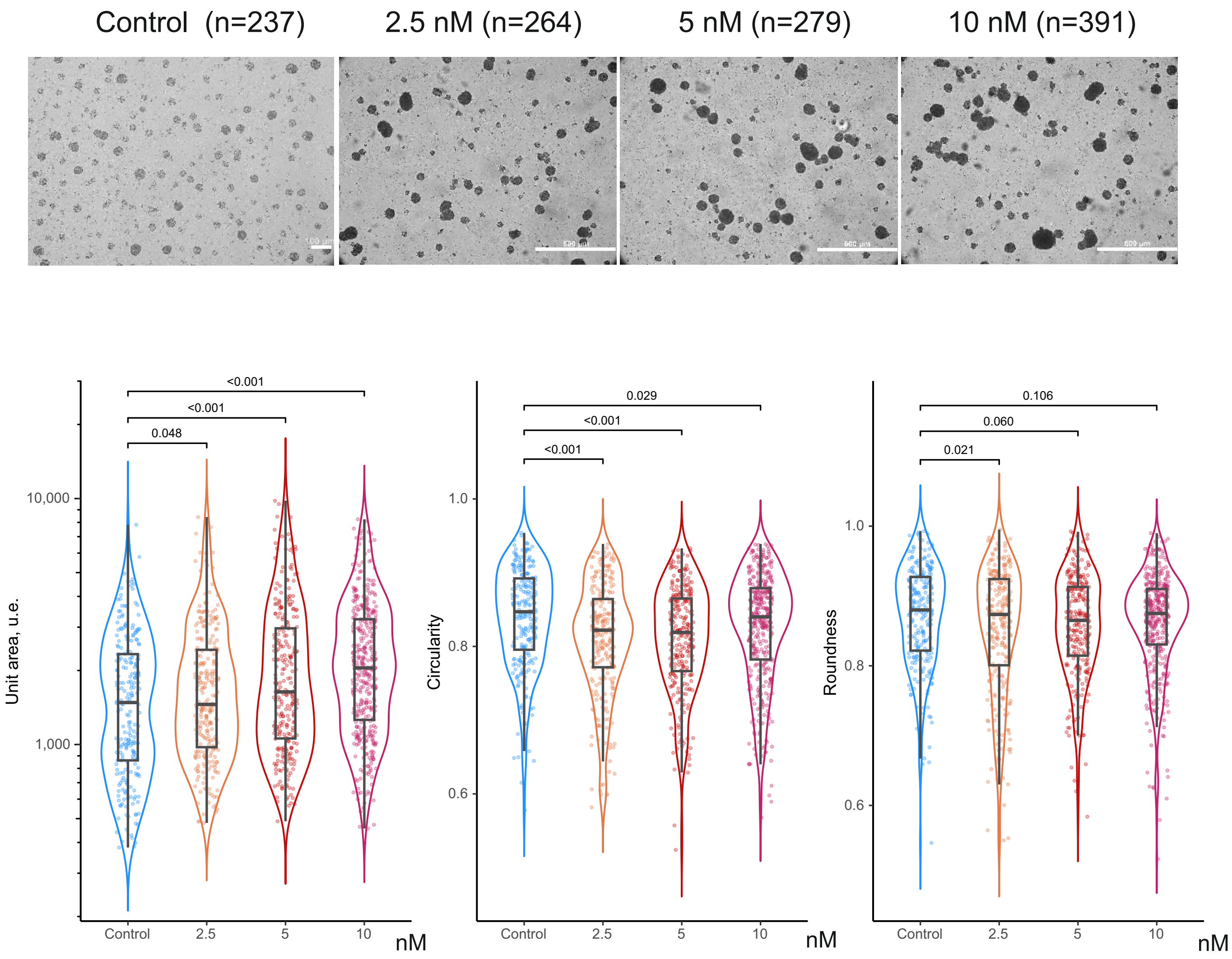

Supplement: Supplementary file 1 [file DataSheet7.PDF]
