# Supplementary material for: Proteomic and transcriptomic signatures of cytoskeletal remodeling during morphogenesis in the basal metazoan Halisarca dujardinii (Porifera)
Source: Front Cell Dev Biol. 2026 Jun 10;14:1829393. doi: 10.3389/fcell.2026.1829393 (PMC13291127; doi:10.3389/fcell.2026.1829393)

Figure S2. Principal component analysis (PCA) of proteomic and transcriptomic datasets. (**A**) PCA plot showing separation of adult sponge body and larval proteome samples based on DIA-LC-MS protein abundance profiles. (**B**) PCA plot of RNA-seq data demonstrating distinct transcriptional clustering of adult sponge body, free-swimming larvae, and 24 h post-dissociation (hpd) cell aggregates. Top 2000 most variable genes were used for RNA-seq PCA plot.

(A)

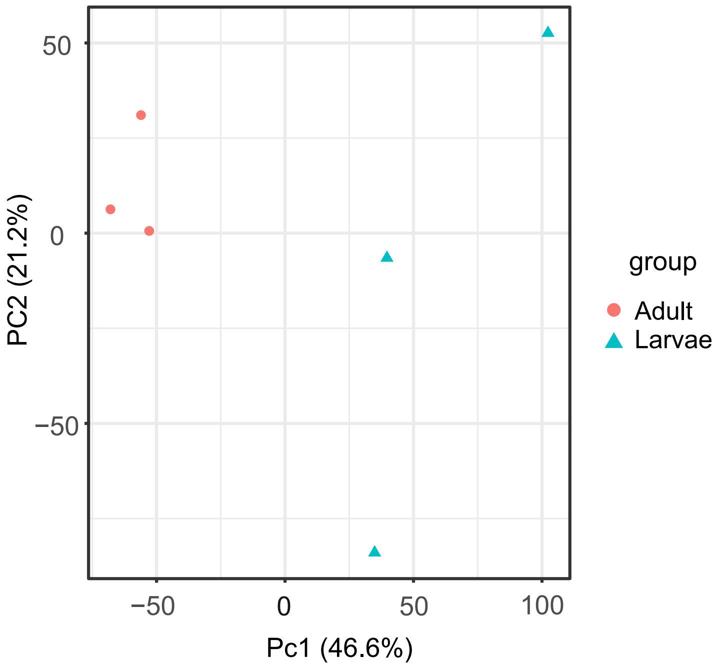

(B)

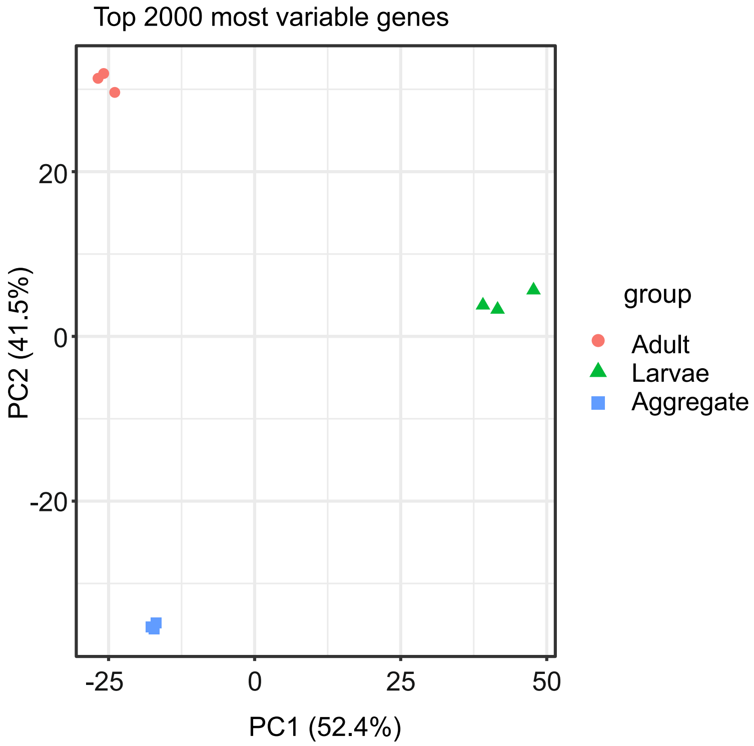

Supplement: Supplementary file 2 [file DataSheet2.PDF]
