# Supplementary material for: Proteomic and transcriptomic signatures of cytoskeletal remodeling during morphogenesis in the basal metazoan Halisarca dujardinii (Porifera)
Source: Front Cell Dev Biol. 2026 Jun 10;14:1829393. doi: 10.3389/fcell.2026.1829393 (PMC13291127; doi:10.3389/fcell.2026.1829393)

Figure S4. Expression of key cytoskeletal genes in *H. dujardinii* across developmental stages.
Transcript levels (CPM) of genes associated with actin- and tubulin-based cytoskeletal functions are shown for adult sponge body, 24 h post-dissociation (hpd) cell aggregates, and larvae. Gene annotations and functional descriptions are provided in Table S6.

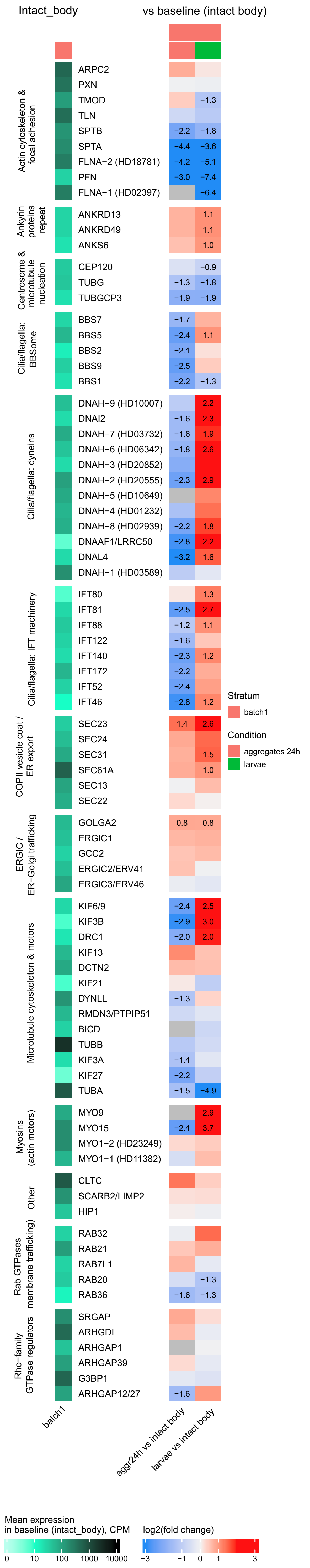

Supplement: Supplementary file 4 [file DataSheet4.PDF]
