# Supplementary material for: Proteomic and transcriptomic signatures of cytoskeletal remodeling during morphogenesis in the basal metazoan Halisarca dujardinii (Porifera)
Source: Front Cell Dev Biol. 2026 Jun 10;14:1829393. doi: 10.3389/fcell.2026.1829393 (PMC13291127; doi:10.3389/fcell.2026.1829393)

Figure S6. Proteasome activity across developmental stages of H. dujardinii (adult, dissociated cells during early reaggregation, and cell aggregates).

(**A**) Live imaging of H. dujardinii cells during early aggregation (1.5 hpd). Active proteasomes were visualized using 1 nM Me4BodipyFL-Ahx3Leu3VS (red). Blue and pink arrows indicate representative single cells and cells within aggregates, respectively. Scale bar, 10 μm.

(**B**) Immunolabeling of actin (ACTBD 11B7; 1:500, green) and live detection of active proteasomes (1 nM Me4BodipyFL-Ahx3Leu3VS, red) in aggregating cells. Arrows highlight actin–proteasome colocalization. Scale bar, 10 μm.

(**C**) Native PAGE analysis of chymotrypsin-like proteasome activity (SLLVY-AMC hydrolysis) in adult sponge (A) and 24 hpd aggregate (Agg) extracts (40 μg protein/lane), with or without 5 nM bortezomib.

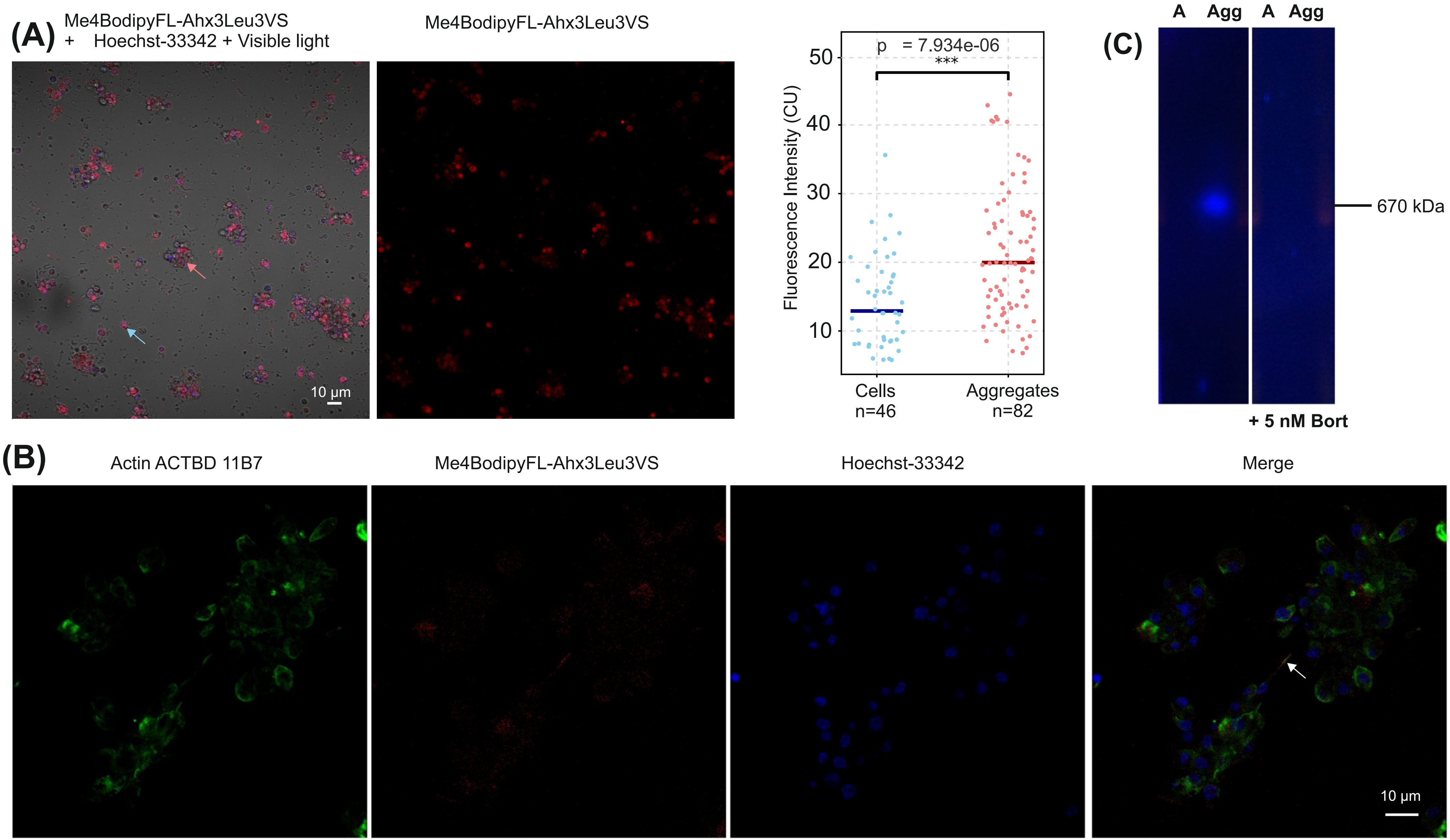

Supplement: Supplementary file 5 [file DataSheet6.PDF]
