# Supplementary material for: Proteomic and transcriptomic signatures of cytoskeletal remodeling during morphogenesis in the basal metazoan Halisarca dujardinii (Porifera)
Source: Front Cell Dev Biol. 2026 Jun 10;14:1829393. doi: 10.3389/fcell.2026.1829393 (PMC13291127; doi:10.3389/fcell.2026.1829393)
Supplement: Supplementary file 7 [file Supplementaryfile1.docx]

**METHODS**

**Sponge body dissociation and cell reaggregation**

The sponges were mechanically dissociated in filtered seawater (FSW) to obtain a homogeneous cell suspension (1x10^7^ cells/ml). To do this, the sponge was cut with sterile scissors and suspended in FSW using a 2 ml syringe. Then, the cell suspension was gently passed through a 40-μm nylon mesh. The filtrate was centrifuged at 300 × g for 5 min at +8°C. Cell viability consistently exceeded 96–98%.

To test whether proteasome activity is required for cell reaggregation, the dissociated *H. dujardinii* cells were treated with selective proteasome inhibitor, bortezomib (2.5, 5, and 10 nM) for the first 24 h of cell reaggregation, and then accessed aggregate morphological organization parameters. The cell survival rate in the presence of bortezomib was previously tested (Table S1). A 3 ml volume of the cell suspension was placed in the wells of a 6-well plate. The dissociated cells were incubated at a temperature of +8^0^C for 24 h in FSW with vehicle (DMSO) or with bortezomib. Aggregates were imaged in transmitted light (Leica DM RXA2 with Olympus DP70) and manually outlined in ImageJ to extract area, perimeter, circularity [4π×Area/Perimeter^2] and roundness [4×Area/(π×MajorAxis^2)] (tables with data are available in (<https://github.com/alexeikotov/Halisarca_dujardinii_mass_spec.git>). Area was log10-transformed to reduce right skew and stabilize variance, improving the validity of mean comparisons. For each inhibitor concentration (2.5, 5, 10 nM) versus control, Welch’s t-tests were performed and p-values adjusted by the Holm method to control family-wise error.

**Transcriptome profiling**

Transcriptome profiling was performed using RNA-seq (BioProject: PRJNA1418124) for three sample types: adult sponge tissue, free-swimming larvae, and 24 hpd cell aggregates (three biological replicates per stage). Paired-end reads were mapped to the genomic assembly using STAR v.2.7.11. Read counts were quantified with featureCounts v.2.14.2 using the parameters “‑M ‑‑fraction.” Principal component analysis was used to assess overall similarity among samples. Differential expression analysis was performed in R using edgeR v.3.42.4 by fitting a generalized linear model with quasi-likelihood testing and using the contrasts “larvae vs. adult” and “aggregates vs. adult”, followed by Benjamini-Hochberg multiple-testing correction. TMM-corrected CPM values were used for downstream analyses. For enrichment analysis, only genes having FDR < 0.05, |log2FC| > 1, and average expression > 3 CPM in at least one of these sample groups.

For downstream analyses and visualizations, candidate genes and proteins of interest were prioritized using a proteomics-first strategy. First, proteins differing between adult and larval samples were identified from the proteomic dataset. Their corresponding transcript abundances were then examined in the RNA-seq dataset. Transcriptomic expression heatmaps of genes of interest were generated using ComplexHeatmap v.2.7.7, using adult expression as the baseline and showing group-averaged log-fold changes (labeling their values only for significant DEGs).

**Functional enrichment analysis**

Functional enrichment analysis was performed in STRING (https://string-db.org/) using default parameters on all differentially represented genes or proteins as a single set. Gene Ontology (GO) biological process categories were used for enrichment analysis, and significance was assessed using STRING’s built-in false discovery rate correction. Protein-protein interaction networks were generated in STRING using the same input protein sets and default network settings.

**Proteomics**

**SDS-PAGE**

Cell lysates of the *H. dujardinii* body tissue, aggregated cells and larvae were extracted in RIPA buffer and protease inhibitor (Sigma). Aliquots containing 80 μg of protein were diluted in sample buffer and maintained for 4 min in a water bath at 95°C. SDS-PAGE electrophoresis (160V) in 10% or 12% polyacrylamide gel was performed followed by Coomassie blue staining. Bands that were the same size as actin (42 kDa) and tubulin (55 kDa) were cut out of the gel for subsequent mass spectrometry analysis.

**Sample preparation**

Reduction, alkylation, and cleavage of proteins were performed as previously described (Kulak et al., 2014), with minor modifications. First, 10 ml of buffer for reduction and alkylation of sodium deoxycholate (SDC) with a pH of 8.5 containing 100 mm TRIS, 1% (by weight) SDC, 10 mm TCEP and 20 mm 2-chloroacetamide were added to 10 ml of the protein sample. Next, the sample was treated with ultrasound in a water bath for 1 min, heated at 85oC for 10 min, then cooled to room temperature and added an equal volume of trypsin solution in a 100 mm TRIS pH 8.5 solution in a 1:50 (by weight) ratio. After overnight incubation at 37oC, the peptides were acidified with 50 μl of 2% trifluoroacetic acid (TFA) mixed with 50 μl of ethyl acetate and loaded into SDB-RPS tubes with two 14-gauge SDB-RPS plugs at the tip. The tubes were centrifuged at 300 g until the entire solution passed through the SDB-RPS filter. The final step took approximately 4 minutes. After washing the StageTips with a 100 µl of 1% TFA/ethyl acetate 1:1 mixture (2 times) and 50 µl of 0.2% TFA, peptides were eluted in a clean tube by 60 µl 60% acetonitrile/5% ammonia mixture using centrifugation at 300 g. The collected material was vacuum-dried and stored at -80^o^C. Before analyses peptides were dissolved in 20 µl of 2% acetonitrile/0.1% TFA and sonicated for 1 min.

**DIA-LC-MS analysis**

DIA-LC-MS analysis was performed as described previously with minor modifications (Kawashima et al., 2022). Peptides were loaded to a home-made trap column 50x0.1 mm, packed with Reprosil-Pur 200 C18-AQ 5 μm (Dr. Maisch), in the loading buffer (2% ACN, 98% H_2_O, 0.1% TFA) at 4 μl/min flow and separated at RT in a home-packed (Kovalchuk et al., 2019) fused-silica column 300x0.1 mm packed with Reprosil-Pur C18-AQ 1.9 μm (Dr. Maisch) into an emitter prepared with P2000 Laser Puller (Sutter, USA). Reverse-phase chromatography was performed with an Ultimate 3000 Nano LC System (Thermo Fisher Scientific), which was coupled to the Orbitrap Tribrid Lumos mass spectrometer (Thermo Fisher Scientific) via a nanoelectrospray source (Thermo Fisher Scientific). Water containing 0.1% (v/v) FA was used as mobile phase A and ACN containing 0.1% FA (v/v), 20% (v/v) H_2_O as mobile phase B. Peptides were eluted from the trap column with a linear gradient: 3-6% B for 5 min, 6-35% B for 53 min, 35-60% B for 4 min, 60% B during 6 min, 60-99% B for 0.1 min, 99% B during 7 min, 99-2% B for 0.1 min at a flow rate of 500 nl/min. MS data was collected in DIA mode. In overlapping window DIA-MS parameters, MS1 spectra were collected in the range of m/z 495-745 at 15,000 resolution to set an AGC target - Standard. MS2 spectra were collected at m/z 200-1800 at 50,000 resolution to set a normalized AGC target of 2000%, a maximum injection time of “auto”, and stepped normalized collision energies of 22, 26, and 30%. The width of the isolation window was set to 4 Da, and overlapping window patterns at m/z 500-740 were used for window placements. The window sizes are listed in Table S2.

**DIA-NN data analysis**

Search parameters of DIA-NN (Demichev et al., 2020) (version 2.2.0) were set as follows: precursor FDR 1%; scan window set to 0; isotopologues and MBR turned on; protein inference at gene level; heuristic protein inference enabled; quantification strategy set to Quant UMS (high precision); neural network classifier single-pass mode (cross-validated); mass accuracy at MS1 and MS2 set to both 0. The protein sequence database was constructed by us based on *H. dujardinii* genome assembly (GCA_054858975.1).The settings for *in silico* library generation from a protein sequence database hd_hybrid_genome_202006_proteins_ann.fasta were as follows: Trypsin/P with maximum 1 missed cleavage; protein N-terminal M excision on; Carbamidomethyl on C as fixed modification; oxidation M, phosphorylation STY and Ac(N-term) as variable modifications; maximum variable modifications 1; peptide length from 7 to 30; precursor charge 1–4; precursor m/z from 300 to 1800; fragment m/z from 200 to 1800.

**Quantitative analysis of protein abundance**

Quantitative analysis of protein abundance was performed in Perseus v.2.0.10 (Tyanova et al., 2016). All runs were analyzed as independent. Specifically, protein intensity values were log2-transformed and retained for downstream analysis if they contained at least three valid values in at least one group. Missing values were then imputed in Perseus from a normal distribution separately for each sample column using a width of 0.3 and a down shift of 2.8. Differential protein abundance between groups was assessed using two-sided Student’s *t*-tests with S0 = 0. Multiple-testing correction was performed using permutation-based false discovery rate (FDR) control with 250 randomizations, and *q*-values were reported. Proteins with |log2FC| > 1 and FDR < 0.05 were considered differentially represented.

**Assessment of Methionine Oxidation**

For the analysis of methionine oxidation, we used several independent biological samples obtained from adult sponges, larvae, and cell aggregates. Sample preparation was performed in parallel processing of all samples (adults, larvae, aggregates) in randomized order to minimize batch effects. To assess methionine oxidation, samples were prepared under denaturing conditions to ensure equal accessibility of all protein regions to potential in vitro artifact oxidation. Preliminary analyses demonstrated that, under our protocol conditions, oxidation was observed only for a subset of methionine residues rather than uniformly across all methionines. For denaturation, samples were heated at 95°C for 3 min in the presence of SDS.

For each sample, a table containing the complete set of identified peptides is provided (<https://github.com/alexeikotov/Halisarca_dujardinii_mass_spec.git>). For further analysis, peptides related only to actins, ferritins and tubulins were used.

For each individual peptide, the degree of methionine oxidation was quantified as the ratio of the ion peak area (Area) of peptides containing oxidized methionine to the total peak area of both oxidized and non-oxidized peptide forms. This calculation was performed separately for each sample.

For subsequent analysis, only peptides consistently detected across all developmental stages (adult sponges, larvae, and aggregates) were selected. Comparisons between stages were based on changes in the ratio of oxidized peptide area to total peptide area (oxidized + non-oxidized), evaluated independently for each methionine residue.

**Cell fixation, immunofluorescent staining and imaging**

Sponge specimens were prefixed with 2% paraformaldehyde freshly diluted in filtered sea water, for 15 min at +4°C, and then extracted for 5 min with cold (−20°C) methanol, which was then gradually washed out with five successive two-fold dilutions by phosphate buffer. The following antibodies were used for immunostaining: mouse monoclonal against α-tubulin DM-1A (T9026, Sigma-Aldrich, Saint Louis, MO, USA), species-specific anti-mouse Ig antibodies (MultiLabeling class) conjugated with fluorochromes FITC (Jackson ImmunoResearch Laboratories (UK) or with Alexa Fluor 633 donkey anti-mouse IgG (Invitrogen/Thermo Fisher Scientific, Waltham, MA, USA). For nuclei staining the Hoechst 33342 (Sigma) was used. For imaging, a Zeiss LSM900 confocal microscope equipped with Zeiss Plan-APOCHROMAT 63×/1.4 Oil DIC objective lens (Zeiss, Oberkochen, Germany) was used (provided by the Moscow State University Development Program). The obtained data were processed using ZEN 3.5 blue edition software (Version 3.5.093.00002) (Zeiss, Oberkochen, Germany).

**Statistical methods**

**Raw data and statistical processing for proteasome activity assays**

The Imagej program was used to analyze the confocal images. The images were analyzed as 8-bit (grayscale) images. The fluorescence level was evaluated as Mean gray for each cell, both for free cells and cells within aggregates. All data are presented in Table S8.

The data was analyzed using the Pandas library (version 2.2.2). Statistical visualization and graphing were performed using the scipy, Matplotlib, and Seaborn libraries for the Python programming language (version 3.12). The statistical significance of the differences between the groups was evaluated using the Mann-Whitney U test.

**Comparative analysis of methionine oxidation**

To evaluate differences in the oxidation ratios of M-sites across various sample categories (Adults, Aggregates, and Larvae), non-parametric statistical tests were employed. The Kruskal-Wallis H-test was conducted to assess overall significant differences among the three independent groups. P-values ​​are provided in Figure S8.

**Comparative analysis of the number of microtubules in cells**

454 adult sponge cells, 544 larval cells, and 124 cells in aggregates were analyzed. In each sample (adult, larva, aggregates) the cells were divided into two groups: those containing microtubules and those without microtubules. Statistical analyses were performed in Python (version 3.12) using the pandas (version 2.2), numpy (version 2.0), and scipy.stats (version 1.16) libraries. Global differences among groups were assessed using Pearson’s χ² test for 3 × 2 contingency tables. The global chi-square test revealed significant differences between groups (χ² = 479.17, df = 2, p = 8.88 × 10⁻105). Post hoc pairwise comparisons were subsequently conducted using Fisher’s exact test. Bonferroni correction was applied to adjust p-values for multiple comparisons.

**Codes**

To study the oxidation of methionins, we used Python codes that were created using the Google Colab service and are available on the website (<https://colab.research.google.com/drive/1ljzWHx3ypQKZ2tLXPJ-tFFJZ4LARDVs9#scrollTo=qJ93wLyYXlcG>).

Demichev, V., Messner, C. B., Vernardis, S. I., Lilley, K. S., and Ralser, M. (2020) DIA-NN: neural networks and interference correction enable deep proteome coverage in high throughput. *Nature methods* 17, 41-44

Tyanova, S., Temu, T., Sinitcyn, P., Carlson, A., Hein, M. Y., Geiger, T., Mann, M., and Cox, J. (2016) The Perseus computational platform for comprehensive analysis of (prote)omics data. *Nature methods* 13, 731-740

Kawashima, Y., Nagai, H., Konno, R., Ishikawa, M., Nakajima, D., Sato, H., Nakamura, R., Furuyashiki, T., and Ohara, O. (2022) Single-Shot 10K Proteome Approach: Over 10,000 Protein Identifications by Data-Independent Acquisition-Based Single-Shot Proteomics with Ion Mobility Spectrometry. *J Proteome Res* 21, 1418-1427

Kovalchuk, S. I., Jensen, O. N., and Rogowska-Wrzesinska, A. (2019) FlashPack: Fast and Simple Preparation of Ultrahigh-performance Capillary Columns for LC-MS. *Molecular & cellular proteomics : MCP* 18, 383-390

Kulak, N. A., Pichler, G., Paron, I., Nagaraj, N., and Mann, M. (2014) Minimal, encapsulated proteomic-sample processing applied to copy-number estimation in eukaryotic cells. *Nature methods* 11, 319-324

Tyanova, S., Temu, T., Sinitcyn, P., Carlson, A., Hein, M. Y., Geiger, T., Mann, M., and Cox, J. (2016) The Perseus computational platform for comprehensive analysis of (prote)omics data. *Nature methods* 13, 731-740
