# Supplementary material for: Proteomic and transcriptomic signatures of cytoskeletal remodeling during morphogenesis in the basal metazoan Halisarca dujardinii (Porifera)
Source: Front Cell Dev Biol. 2026 Jun 10;14:1829393. doi: 10.3389/fcell.2026.1829393 (PMC13291127; doi:10.3389/fcell.2026.1829393)
Supplement: Supplementary file 8 [file Table1.docx]

Table S1. Cell viability in the presence of different concentrations of bortezomib, assessed by incubation with trypan blue.

| Concentration | 0 nM | 2.5 nM | 5 nM | 10 nM |
| --- | --- | --- | --- | --- |
| Total cell number | 531 | 702 | 161 | 250 |
| Live cell number | 485 | 610 | 109 | 168 |
| Viability (%) | 91.3 | 86.9 | 67.7 | 67.2 |
