# Supplementary material for: Proteomic and transcriptomic signatures of cytoskeletal remodeling during morphogenesis in the basal metazoan Halisarca dujardinii (Porifera)
Source: Front Cell Dev Biol. 2026 Jun 10;14:1829393. doi: 10.3389/fcell.2026.1829393 (PMC13291127; doi:10.3389/fcell.2026.1829393)
Supplement: Supplementary file 9 [file Table7.doc]

Table S7. Extended LC-MS/MS data for *Halisarca dujardinii* native protein complexes (A) and SDS samples (B). The analysis of methionine oxidation in SDS samples (B) was carried out separately as described in the Supplementary methods (Figure S), 10 samples were analyzed (PRIDE: PXD066419).

A.

| Name | Genbank ID | #Peptides, Larva | PTM, Larva | #Peptides, Aggr | PTM, Aggregates | #Peptides, Adult | PTM, Adult |
| --- | --- | --- | --- | --- | --- | --- | --- |
| ARHGDI, RHOGDI Rho GDP dissociation inhibitor | PX991082 | 2 | Deamidation (+0.98) Q157 | 6 | Oxidation (+15.99) M160 | 4 | Carbamidomethylation (+57.02) C106 |
| ARPC2, actin related protein 2-3 complex subunit 2 | ON088640 | - |  | 11 | Oxidation (+15.99) M148 | - |  |
| SEC31, protein transport protein SEC31 | PX991103 | 3 |  | - |  | - |  |
| CLTC, clathrin heavy chain | PX991133 | 5 | Carbamidomethylation (+57.02) C1529 | 37 | Oxidation (+15.99) M1121; Deamidation (+0.98) N240; Carbamidomethylation (+57.02) C738 | - |  |
|  |  |  |  |  |  |  |  |
| TUBA6, tubulin_alpha 6 | MT451965 | - |  | - |  | 8 | Oxidation (+15.99) M398 |
| TUBA7, tubulin alpha 7 | MT451966 | 2 |  | - |  | 5 | Oxidation (+15.99) M398 |
| TUBB, tubulin beta | OM892945 | 10 | Oxidation (+15.99) M257, M388;  Carbamidomethylation (+57.02) C12;  Deamidation (+0.98) N337 | 9 | Oxidation (+15.99) M267, M293, M363;  Deamidation (+0.98) N347 | 15 | Oxidation (+15.99) M73, V164, M257, M330;  Carbamidomethylation (+57.02) C12, C303, C354 |
| HdA1/2/3, Actin 1/2/3 | MT451954  MT451955  MT451956 | 10 | Oxidation (+15.99) M191, M326  Acetylation (+42.01) G2  Carbamidomethylation C18, C286  Methylation (+14.02) K19 | 36 | Oxidation (+15.99) M45, M48, M306  Acetylation (+42.01) Deamidation (+0.98) Q42, Q60, N112, N253, N297  Carbamidomethylation C258 | 58 | Oxidation (+15.99) M17, M45, M48, M306, M326  Acetylation (+42.01) G2, Q361  Deamidation (+0.98) N13, Q42, Q60, Q361  Carbamidomethylation C18, C258, C286 |
| HdA6, Actin 6 | MT451959 | 2 |  | 3 | Carbamidomethylation (+57.02) M48;  Oxidation (+15.99) M48 | 7 | Oxidation (+15.99) M86 |
| HdF1a/b, Ferritin 1 | MK520983  MT423816 | 47 | Oxidation (+15.99) M23, M45, M67, M97, M114;  Deamidation (+0.98) N8, N18, Q70, N71, Q80, N108, Q109, Q140, Q144, Q151;  Acetylation (+42.01) K65, F66, D106;  Methylation (+14.02) E7;  Phosphorylation (+79.97) (+79.97) Y134 | 136 | Oxidation (+15.99) M23, M45, M67, M97, M114, M126;  Deamidation (+0.98) Q70, N71, Q98, Q102, N108, Q109, Q125, N133, Q138, Q144, Q151;  Carbamidomethylation (+57.02) C127, N133;  Acetylation (+42.01) D88, D106, I117, Q144;  Ubiquitin (+114.04) S92, C127, T150, K153;  Acetylation (+42.01) S92, V155;  Methylation K84, K87 | 70 | Oxidation (+15.99) M45, M67, M97, M114, M126;  Deamidation (+0.98) N8, Q70, N71, Q80, Q98, C127, N133, Q138;  Acetylation (+42.01) E7, F66, S92, I117;  Carbamidomethylation (+57.02) Q125, C127;  Phosphorylation (+79.97) S117 |
| HdF2, Ferritin 2 | MT423817 | 2 |  | 8 | Oxidation (+15.99) M68, M127; Deamidation (+0.98) Q71, Q88; Carbamidomethylation (+57.02) C149 | 17 | Deamidation (+0.98) N72, N14; Oxidation (+15.99) M98, M104; Carbamidomethylation (+57.02) C149 |

B.

| Name | Genbank mRNA ID | #Peptides, Larva | PTM, Larva | #Peptides, Aggregates | PTM, Aggregates | #Peptides, Adult | PTM, Adult |
| --- | --- | --- | --- | --- | --- | --- | --- |
| ARPC2, actin related protein 2-3 complex subunit 2 | ON088640 | 16 | Carbamidomethylation (+57.02) C261 | 12 | Oxidation (+15.99) M148, M178 | 14 | Oxidation (+15.99) M148, M178 |
| TLN, talin | PX991087 | - |  | 10 | Oxidation (+15.99) M1765, M1861 | 5 |  |
| CLTC, clathrin heavy chain | PX991133 | - |  | 2 | Oxidation (+15.99) M73, M191 | - |  |
|  |  |  |  |  |  |  |  |
| TUBA1/2/3/4, tubulin alpha 1/2/3/4 | MT451960  MT451961 MT451962  MT451963 | 13 | Oxidation (+15.99) M93, M114, M159;  Carbamidomethylation (+57.02) C347;  Phosphorylation (+79.97) Y312 | 32 | Oxidation (+15.99) M93, M114, M159;  Deamidation (+0.98) N101;  Acetylation (+42.01) Q91;  Ubiquitin (+114.04) T94 | 124 | Oxidation (+15.99) M93, M114, M159;  Deamidation (+0.98) N216;  Acetylation (+42.01) D113, Y312, A374;  Ubiquitin (+114.04) C376;  Phosphorylation (+79.97) S379;  Methylation (+14.02) K304, K352 |
| TUBA5, tubulin alpha 5 | MT451964 | 3 |  | 18 | Phosphorylation (+79.97) Y312 | 87 | Acetylation (+42.01) A374;  Ubiquitin (+114.04) C376;  Phosphorylation (+79.97) S379;  Methylation (+14.02) K304, K352 |
| TUBA6, tubulin alpha 6 | MT451965 | 3 |  | 10 |  | 69 | Acetylation (+42.01) A374;  Ubiquitin (+114.04) C376;  Phosphorylation (+79.97) S379 |
| TUBA7, tubulin alpha 7 | MT451966 | 3 |  | 12 | Deamidation (+0.98) N101 | 48 |  |
| TUBA8, tubulin alpha 8 | MT451967 | 2 | Acetylation (+42.01) Q85 | 6 |  | 100 | Methylation (+14.02) R390 |
| TUBA9, tubulin alpha 9 | MT451968 | 2 |  | 5 |  | 39 | Oxidation (+15.99) M113, M202, M301, M312, M398, M412, M424;  Acetylation (+42.01) W387 |
| TUBA10, tubulin alpha 10 | OM982448 | 6 | Phosphorylation (+79.97) Y312 | 18 | Oxidation (+15.99) M398; Deamidation (+0.98) N101 | 91 | Acetylation (+42.01) Y24, D113, Y312, A374;  Ubiquitin (+114.04) C376;  Phosphorylation (+79.97) S379;  Methylation (+14.02) K304, K352 |
| TUBB, tubulin beta | OM892945 | 28 | Oxidation (+15.99) M73, M164, M267, M316, M330, M363;  Carbamidomethylation (+57.02) C303, C354;  Deamidation (+0.98) N347;  Acetylation (+42.01) L389 | 42 | Oxidation (+15.99) M73, M164, M267, M316, M330, M363;  Carbamidomethylation (+57.02) C12, C303;  Ubiquitin (+114.04) T160;  Methylation (+14.02) K103; R306 | 161 | Oxidation (+15.99) M73, M164, M267, M316, M330, M363;  Carbamidomethylation (+57.02) C12, C303, C354;  Acetylation (+42.01) Y159, I163, L317, E325, L353, N398;  Ubiquitin (+114.04) C12, T107;  Phosphorylation (+79.97) S322;  Methylation (+14.02) K19, R46, R341 |
| HdA1/2/3, Actin 1/2/3 | MT451954  MT451955  MT451956 | 393 | Oxidation (+15.99) M45, M48, M120, M228, M270, M284, M314, M326, M356;  Acetylation (+42.01) G2, A20  Deamidation (+0.98) N13, Q42, Q60, Q361  Carbamidomethylation (+57.02) C18, C218, C258, C286;  Methylation (+14.02) K19, H74;  Ubiquitin (+114.04) K51, T67, S200, C286, T352;  Ubiquitination (+383.23) K214;  Phosphorylation (+79.97) S266, T319 | 336 | Oxidation (+15.99) M45, M48, M120, M228, M270, M284, M314, M326, M356;  Acetylation (+42.01) G2, A20  Deamidation (+0.98) Q42, Q60, N112, N93, N297  Carbamidomethylation (+57.02) C18, C218, C258, C286;  Methylation (+14.02) K19, H74;  Ubiquitin (+114.04) K51, C286 | 323 | Oxidation (+15.99) M45, M48, M120, M228, M270, M284, M314, M326, M356;  Acetylation (+42.01) G2, A20  Deamidation (+0.98) N13, Q42, Q60, Q361  Carbamidomethylation (+57.02) C18, C218, C258, C286;  Methylation (+14.02) K19, H74;  Ubiquitin (+114.04) K51, S61, K62, S200, C286, K316, S359;  Ubiquitination (+383.23) K19;  Phosphorylation (+79.97) S15, S53, S200, Y219, T319, T352 |
| HdA6, Actin 6 | MT451959 | 65 | Oxidation (+15.99) M48, M51, M86, M329;  Carbamidomethylation (+57.02) C37, C56, C289;  Deamidation (+0.98) Q45;  Methylation (+14.02) R43;  Ubiquitination (+383.23) K88;  Ubiquitin (+114.04) T93 | 51 | Oxidation (+15.99) M48, M51, M86, M329; | 22 | Oxidation (+15.99) M48, M51, M86, M329;  Carbamidomethylation (+57.02) C56 |
| HdF1a/b, Ferritin | MK520983  MT423816 | 10 | Oxidation (+15.99) M45, M67, M97, M114, M126 | 7 | Oxidation (+15.99) M45, M67, M97, M114, M126 | 14 | Oxidation (+15.99) M45, M67, M97, M114, M126 |
