# Supplementary material for: Proteomic and transcriptomic signatures of cytoskeletal remodeling during morphogenesis in the basal metazoan Halisarca dujardinii (Porifera)
Source: Front Cell Dev Biol. 2026 Jun 10;14:1829393. doi: 10.3389/fcell.2026.1829393 (PMC13291127; doi:10.3389/fcell.2026.1829393)

Figure S3. Gene Ontology (GO) enrichment analysis of biological processes in *H. dujardinii* adult sponge body (A) and larvae (B).
Enriched terms are color-coded by functional category: red, sensory organ morphogenesis (GO:0090596, FDR < $3.4 \times 10^{\ 4}$); green, negative regulation of phosphorylation (GO:0042326, FDR = 0.02); blue, embryonic development (GO:0009790, FDR = 0.04). Panel **A** shows processes enriched in adult-specific proteins; panel **B** shows larval-specific enrichments. Gene lists and full enrichment statistics for stage-specific proteins are provided in Table S4.

(A)

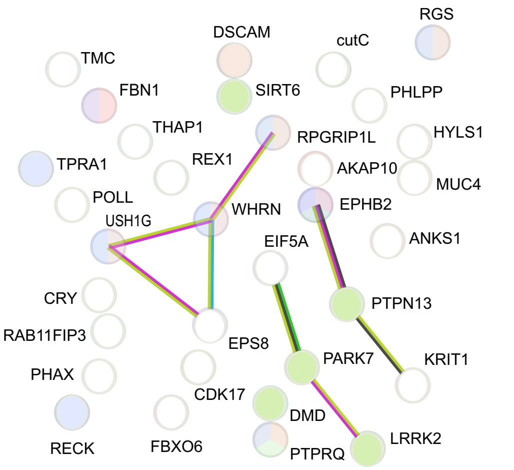

(B)

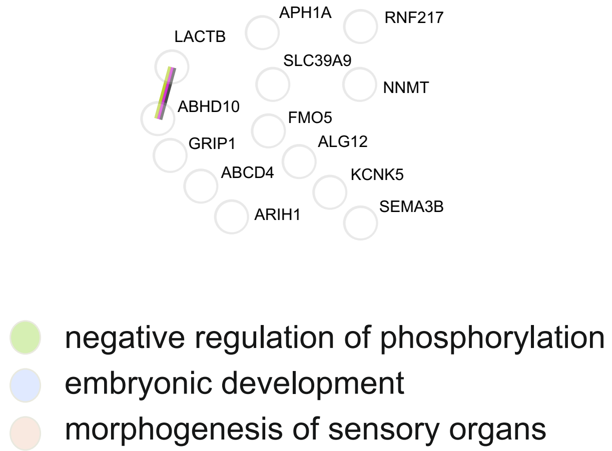

Supplement: Supplementary file 10 [file DataSheet3.PDF]
