# Supplementary material for: Proteomic and transcriptomic signatures of cytoskeletal remodeling during morphogenesis in the basal metazoan Halisarca dujardinii (Porifera)
Source: Front Cell Dev Biol. 2026 Jun 10;14:1829393. doi: 10.3389/fcell.2026.1829393 (PMC13291127; doi:10.3389/fcell.2026.1829393)

Figure S1.The specimens of Arctic sponge *H. dujardinii*.
(**A**) Sampling site of *H. dujardinii*. (**B**) Adult individual on Fucus. (**C,D**) Larva. (**E**) Cell aggregates. (**F-J**) Recovery of the sponge from the cell suspension. The dissociation of the sponge and reaggregation were performed at a temperature of +6-+8 degrees, as described in the supplementary methods. (**F**) Initial multicellular aggregates, (**G**) early-stage primmorph, (**H**) primmorph, (**I,J**) fully developed juvenile sponges. The osculum is shown by an arrow.

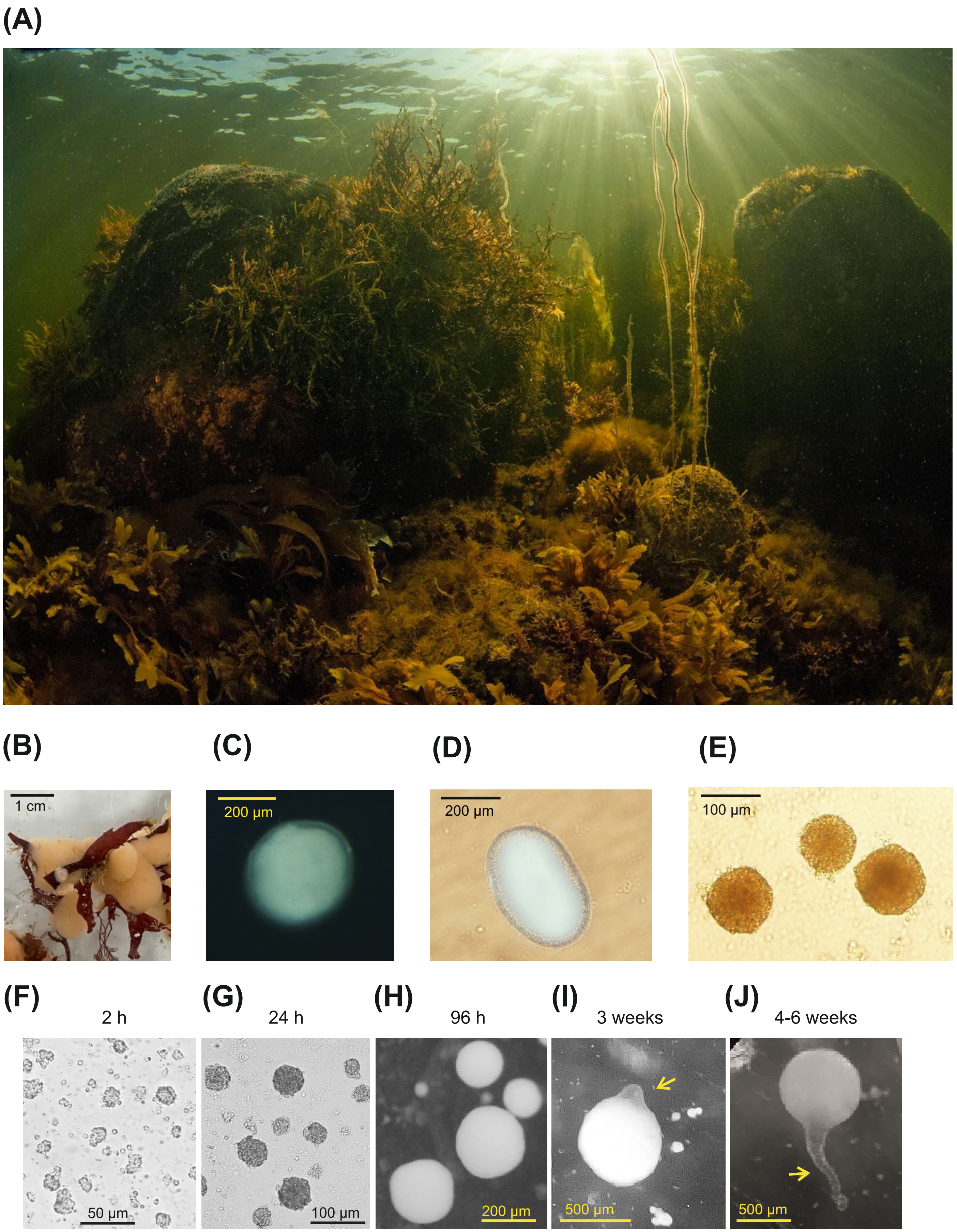

Supplement: Supplementary file 12 [file DataSheet1.PDF]
