# Supplementary material for: Proteomic and transcriptomic signatures of cytoskeletal remodeling during morphogenesis in the basal metazoan Halisarca dujardinii (Porifera)
Source: Front Cell Dev Biol. 2026 Jun 10;14:1829393. doi: 10.3389/fcell.2026.1829393 (PMC13291127; doi:10.3389/fcell.2026.1829393)

Figure S5. Single-cell RNA-seq deconvolution analysis of stage-specific cellular composition. Single-cell RNA-seq data are publicly accessible via Zenodo (doi.org/10.5281/zenodo.14981466). (**A**) Cell-type deconvolution of bulk RNA-seq samples from adult sponge body, free-swimming larvae, and 24 h post-dissociation (hpd) cell aggregates. Clusters 0 and 2 were combined due to similar expression profiles and unstable proportion predictions; clusters 19 and 20 were excluded owing to insufficient marker gene stability. (**B**) Integration of deconvolution results across biological replicates. (**C**) Expression of genes involved in actin and tubulin cytoskeletal organization, as well as ferritin subunits (*HdF1a/b*), across annotated cell clusters.

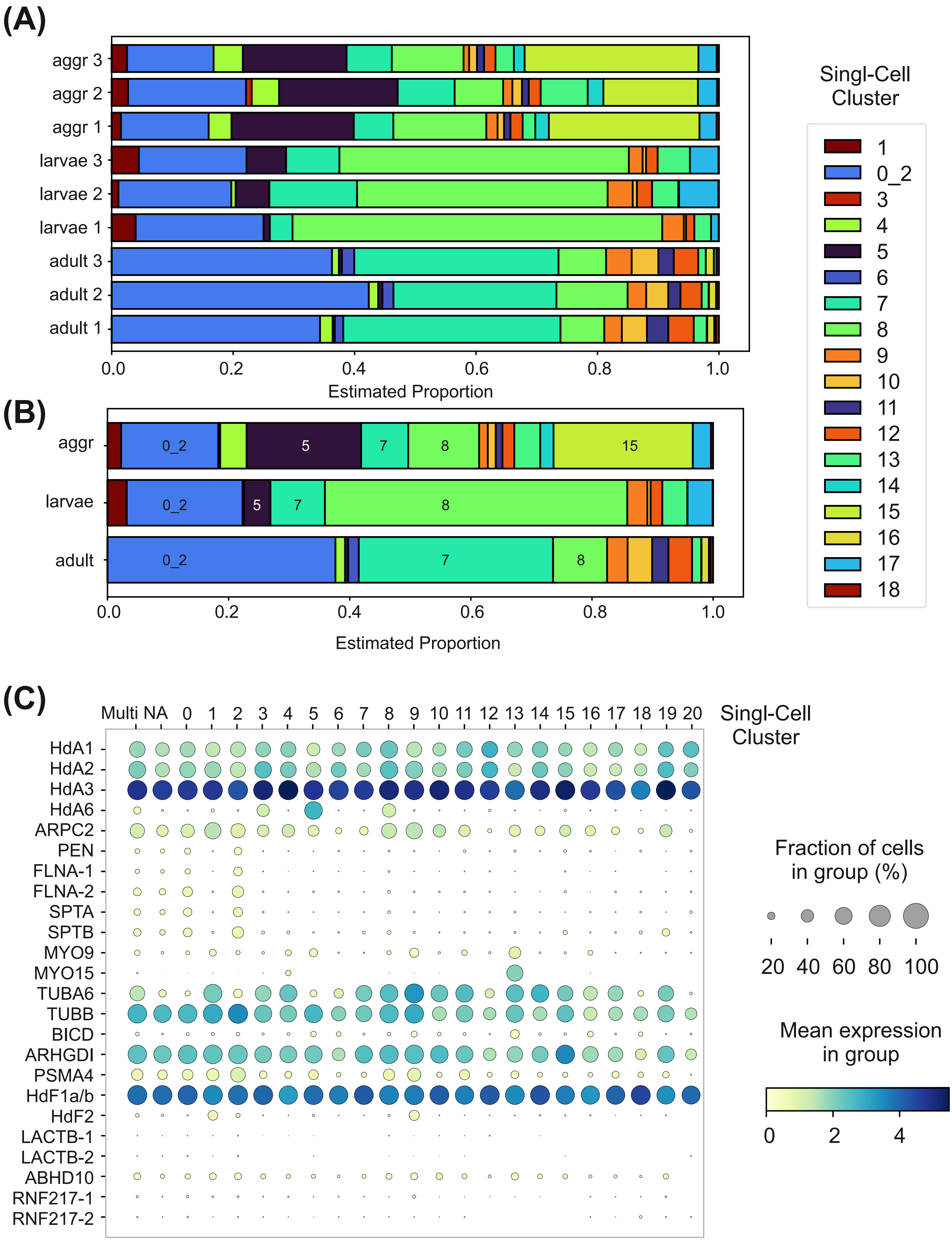

Supplement: Supplementary file 13 [file DataSheet5.PDF]
