# Supplementary material for: Proteomic and transcriptomic signatures of cytoskeletal remodeling during morphogenesis in the basal metazoan Halisarca dujardinii (Porifera)
Source: Front Cell Dev Biol. 2026 Jun 10;14:1829393. doi: 10.3389/fcell.2026.1829393 (PMC13291127; doi:10.3389/fcell.2026.1829393)

**Figure S8. Stage-specific methionine oxidation profiles of cytoskeletal proteins in *H. dujardinii*.**
Methionine oxidation was quantified for actin isoforms (HdA1/2/3, accession QSX72278; HdA6, QSX72283), α-tubulins (α1, QSX72284; α2, QSX72285), β-tubulin (WAQ15573), and ferritin subunits (HdF1a/b, QEH04776) across adult sponge body, free-swimming larvae, and 24 h post-dissociation (hpd) cell aggregates. For each peptide containing methionine, the extent of oxidation was calculated as the ratio of the ion peak area of the oxidized form to the total peak area (oxidized + non-oxidized) within each biological replicate. Only peptides consistently detected across all three developmental stages were included in the comparative analysis. Stage-specific differences were assessed by comparing oxidation ratios for each methionine residue independently.

## actin 1/2/3

```
                                                                      45  48
  1 MGDEDVAALV VDNGSGMCKA GFAGDDAPRA VFPSIVGRPR HQGVMVGMGQ KDSYVGDEAQ SKRGILTLKY PIEHGIVTNW DDMEKIWHHT FYNELRVAPE

101 EHPVLLTEAP LNPKANREKM TQIMFETFNT PAMYVAIQAV LSLYASGRTT GIVFDSGDGV SHTVPIYEGY ALPHAILRLD LAGRDLTDYL MKILTERGYS
                                 228
201 FTTTAEREIV RDIKEKLCYV ALDFEQEMQT AASSSSLEKS YELPDGQVIT IGNERFRCPE ALFQPSFLGM ESAGIHETTY NSIMKCDVDI RKDLYANTVL
                               326                                  356
301 SGGSTMFPGI ADRMQKEITA LAPPTMKIKI IAPPERKYSV WIGGSILASL STFQQMWISK QEYDESGPSI VHRKCF
```

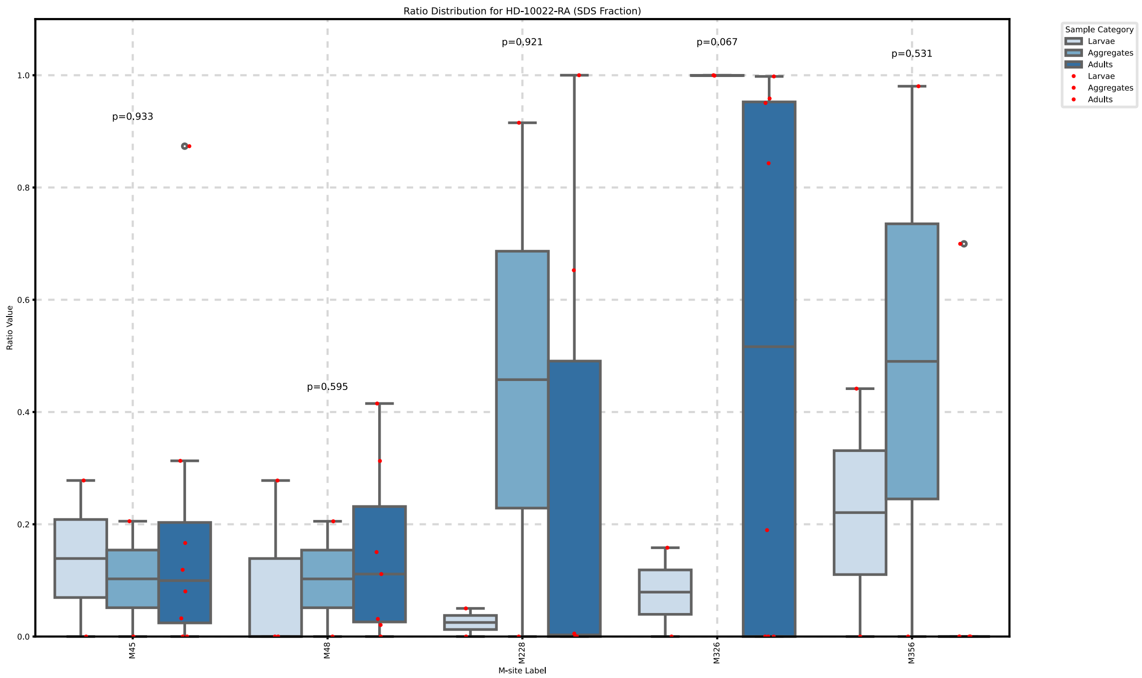

# actin 6

48 51 86

1 MSDSDDEGSA PIVIDNGSGM MKAGYGGEDA PQVVFPCLVG NPRHQGVMVG MGEKDCYVGD EAESKRGILT ISYPVEHGIV TNWDDMEKIW HHTVYNELRA

101 DPEEHPILVT EAPLNPKANR ERMISILFET FGVPAAYVAI QAVMALYSAG RTTGIVCDSG DGVTHTVPVY EGYALPHAIG RLDLAGRDIT HYLTKILTER

201 GYSFTTTAEL EIIKDMKEKL SFVSTDFEKD MEDSGKQSRF EKTYELPDGQ VITIANERFR APESMFKPAF LGRESAGVHE LAYTSIMKCD IDIRKDLYAN

329

301 IVLSGGNTMY AGMAERMQRE IADLTPPSMK VKVIAPPERK FSVWIGASIL SNLSTFQQMW ISREEFDEAG PGIVHKKCF

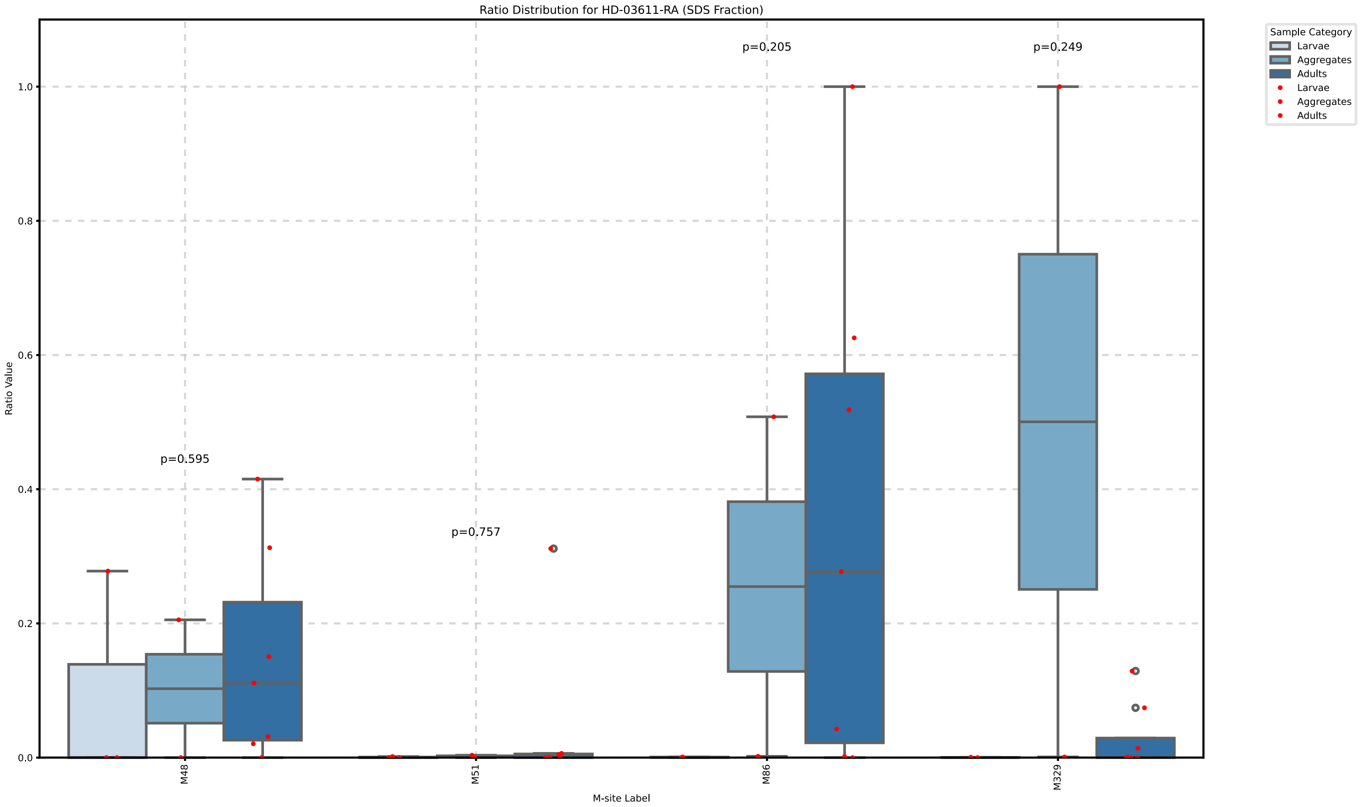

# tubulin alpha 1

1 MRECISIHVG QAGCQIGNAC WELYCLEHGI TPDGMMPSDT TLGGGNDSFN TFFSETGAGK HVPRCVFVDL EPSVVDEIRT GTYRQLFHPD QLITGKEDAA

159

101 NNYARGHYTV GKEYIDRIVD RIRILADQCT GLQGFLVFHS FGGGTGSGLT SLLMERLSMD YGKKSKLEFA VYPAPHISTA MVEPYNAILT THTTLEHTDC

201 AFMVDNEAIY DICRRNLDVE RPSYSNLNRL ISQIVSSITA SLRFEGSLNV DLTEFQTNLV PYPRIHFPLV TYAPIISAEK AQHEQFTVAD ITNACFETHN

301 QMVKCDPHAG KYMACCLLYR GDVVPKDVNT SIAAIKAKRD IQFVDWCPTG FKVGINYKSP TAVPGGDLAP VKRAVCMLSN TTAIAEAWQR LDHKFDLMYA

401 KRAFVHWYVG EGMEEGEFQE AREDMAALEK DYEEVGADTP EGDEDAEEEY

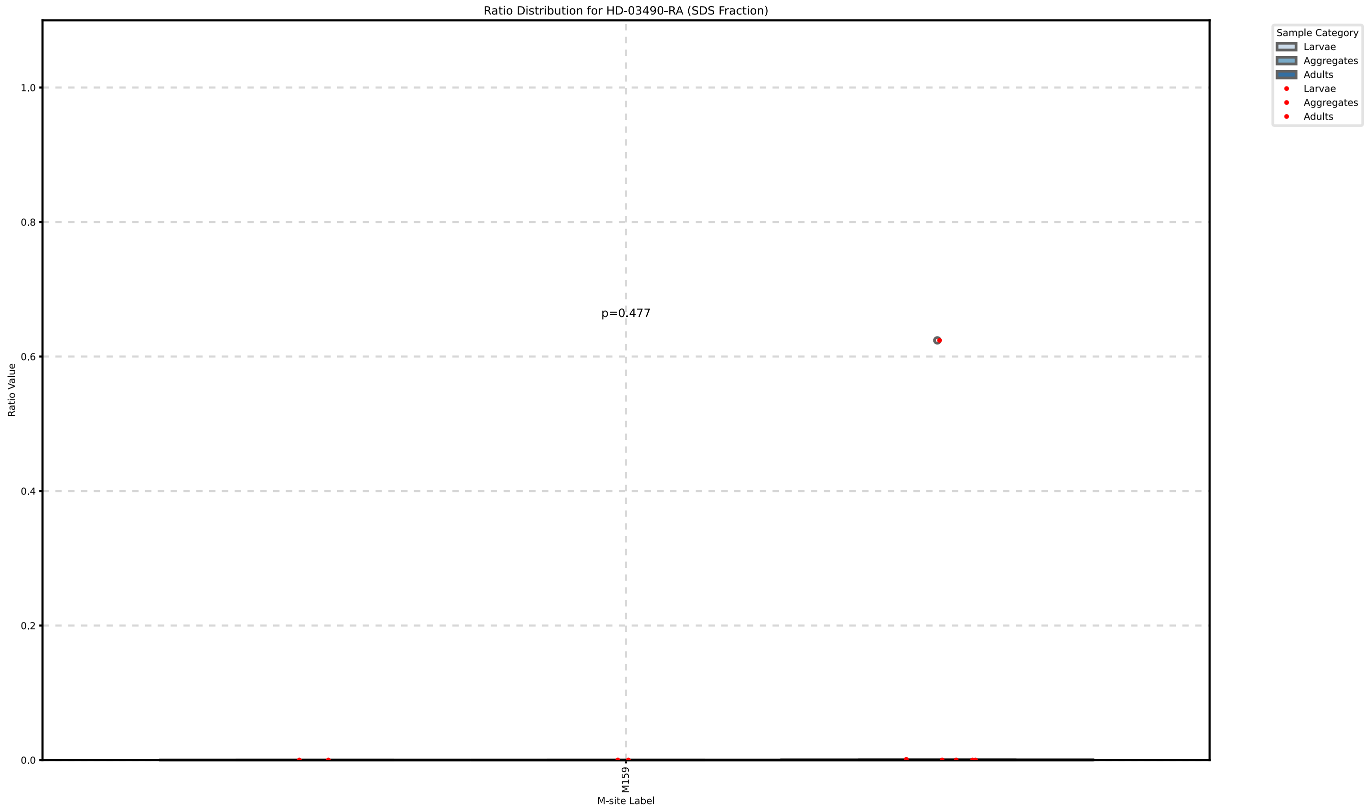

# tubulin alpha 2

```
                                                                                                       93
1   MRECISIHVG QAGVQIGNAC WELYCLEHGI QPDGQMPSDK TIGGGDDSFN TFFSETGAGK HVPRAVFVDL EPTVVDEVRT GTYRQLFHPE QLMTGKEDAA
                  114
101 NNYARGHYTV GKDMIDLVLD RIRKLADQCT GLQGFLVFHS FGGGTGSGFA SLLLERLSVD YGKKSKLEFA IYPAPQISTA VVEPYNSILT THTTLEHSDC
201 AFMVDNEAVY DICRRNLDIE RPTYTNLNRL MGQIVSSITA SLRFDGALNV DLTEFQTNLV PYPRIHFPLV TYAPIISAEK AYHEQLSVAE ITNACFEPAN
301 QMVKCDPRHG KYMSCCMLYR GDVVPKDVNA AIATIKTKRT IQFVDWCPTG FKVGINYQPP TVVPGGDLAK VQRAVCMLSN TTAIAEAWAR LDHKFDLMYA
401 KRAFVHWYVG EGMEEGEFSE AREDLAALEK DYEEVGMDTV EGEGEGEGED EY
```

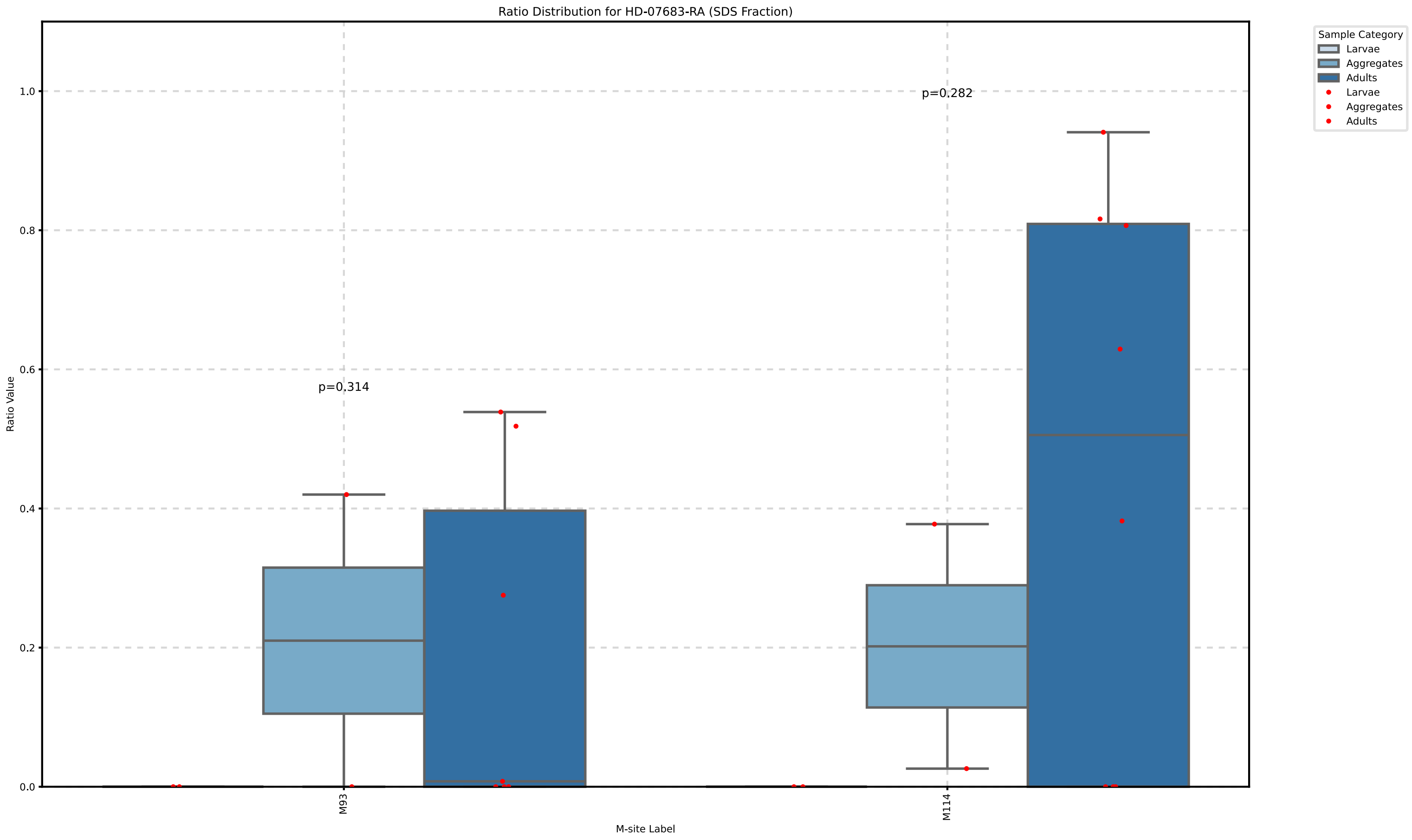

# tubulin beta

```
  1 MREIVHLQAG QCGNQIGAKF WEVISDEHGI DPTGTYHGDS DLQLERINVY YNEATGGKYV PRAVLVDLEP GTMDSVRSGP FGQIFRPDNF VFGQSGAGNN
                                                                     164
101 WAKGHYTEGA ELVDSVLDVV RKEAEGCDCL QGFQLTHSLG GGTGSGMGTL LISKIREEYP DRIMNTFSVV PSPKVSDTVV EPYNATLSVH QLVENTDETY
201 CIDNEALYDI CFRTLKLTTP TYGDLNHLVS ATMSGVTTCL RFPGQLNADL RKLAVNMVPF PRLHFFMPGF APLTSRGSQQ YRALTVPELT QQMFDAKNMM
                                   330
301 AACDPRHGRY LTVAAMFRGR MSMKEVDEQM LNVQNKNSSY FVEWIPNNVK TAVCDIPPRG LKMSATFIGN STAIQELFKR ISEQFTAMFR RKAFLHWYTG
401 EGMDEMEFTE AESNMNDLVS EYQQYQDATA EDEGAEDEDE EEEEA
```

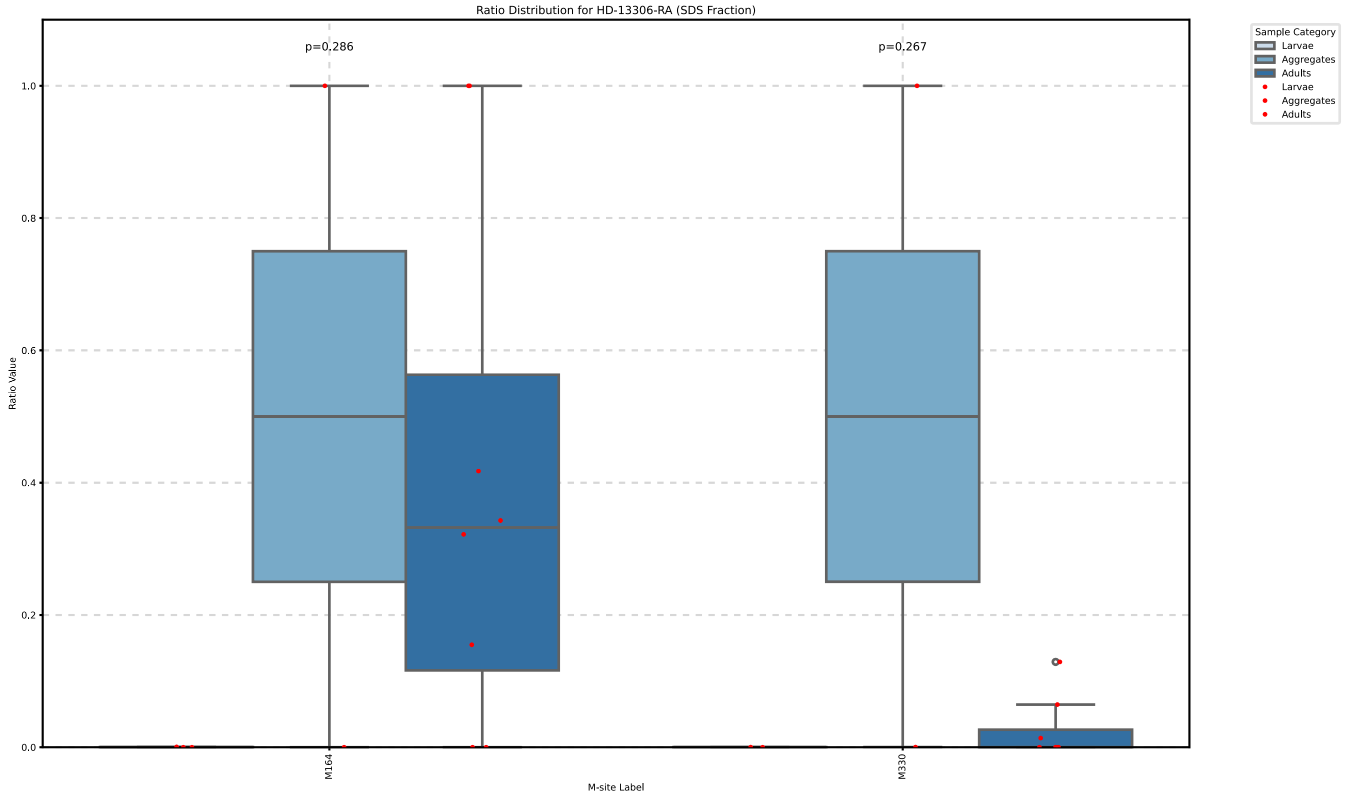

# ferritin 1/2

45 67 97

1 MASQVRENYH EESEAAVNKQ INMELYAFYT YTSLAFYFDR DDIAMSGFKD FFLKSADEEY DHAKKFMAFQ NERGGRIVLQ DVKKPAKDEW GSGAEAMQAA

114

101 LQLEKDVNQA LLDMHKISDS HGDAQMCDFI EANYLTEQTQ AIKQLGDYVT QLKKVGTGHG EWHFQKDLA

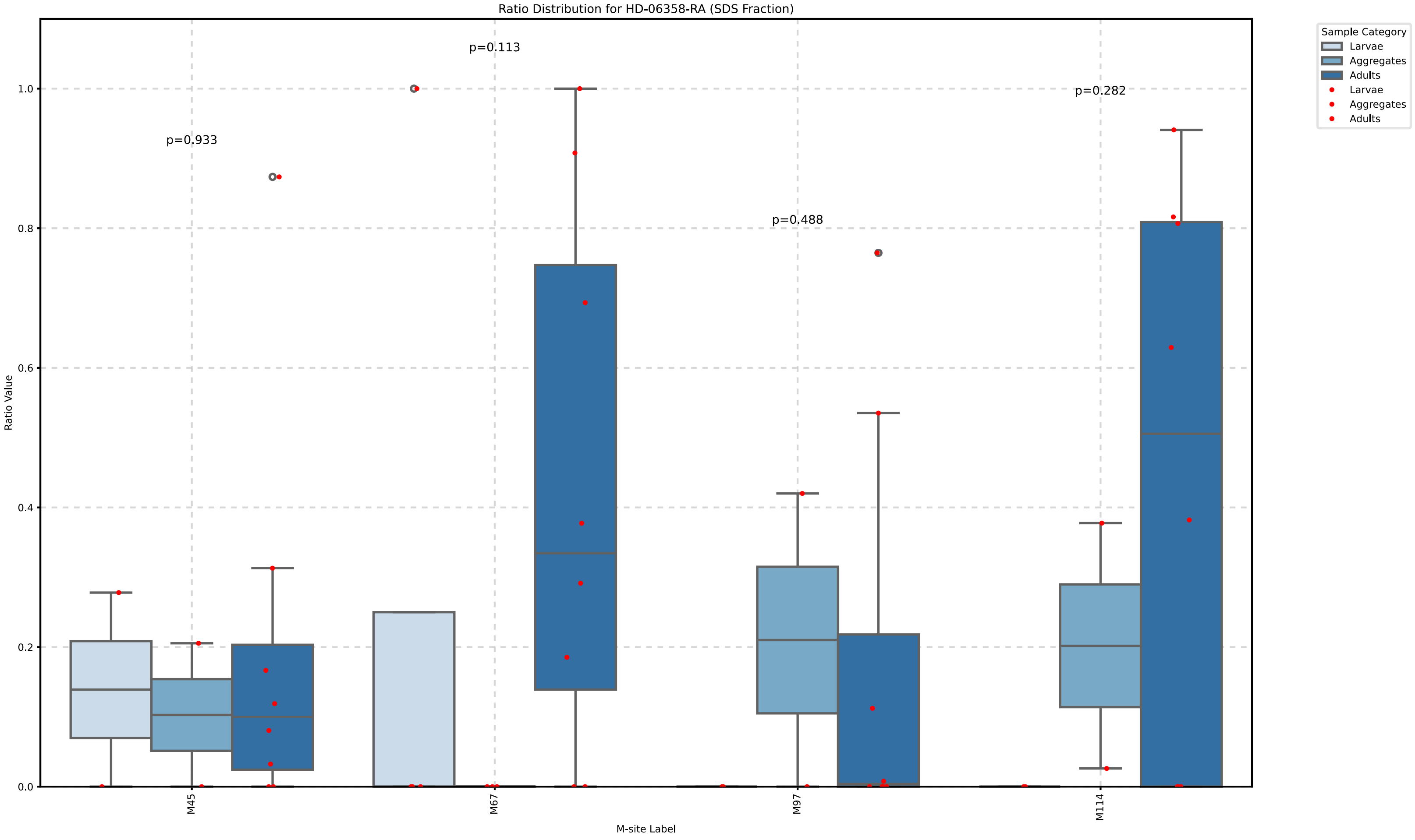

Supplement: Supplementary file 15 [file DataSheet8.PDF]
